# Enhanced perfusion following exposure to radiotherapy: A theoretical investigation

**DOI:** 10.1371/journal.pcbi.1011252

**Published:** 2024-02-16

**Authors:** Jakub Köry, Vedang Narain, Bernadette J. Stolz, Jakob Kaeppler, Bostjan Markelc, Ruth J. Muschel, Philip K. Maini, Joe M. Pitt-Francis, Helen M. Byrne

**Affiliations:** 1 School of Mathematics and Statistics, University of Glasgow, Glasgow, United Kingdom; 2 Mathematical Institute, University of Oxford, Oxford, United Kingdom; 3 Laboratory for Topology and Neuroscience, École Polytechnique Fédérale de Lausanne, Lausanne, Switzerland; 4 Cancer Research UK and MRC Oxford Institute for Radiation Oncology, Department of Oncology, University of Oxford, Oxford, United Kingdom; 5 Department of Experimental Oncology, Institute of Oncology Ljubljana, Ljubljana, Slovenia; 6 Department of Computer Science, University of Oxford, Oxford, United Kingdom; University of Connecticut School of Medicine, UNITED STATES

## Abstract

Tumour angiogenesis leads to the formation of blood vessels that are structurally and spatially heterogeneous. Poor blood perfusion, in conjunction with increased hypoxia and oxygen heterogeneity, impairs a tumour’s response to radiotherapy. The optimal strategy for enhancing tumour perfusion remains unclear, preventing its regular deployment in combination therapies. In this work, we first identify vascular architectural features that correlate with enhanced perfusion following radiotherapy, using *in vivo* imaging data from vascular tumours. Then, we present a novel computational model to determine the relationship between these architectural features and blood perfusion *in silico*. If perfusion is defined to be the proportion of vessels that support blood flow, we find that vascular networks with small mean diameters and large numbers of angiogenic sprouts show the largest increases in perfusion post-irradiation for both biological and synthetic tumours. We also identify cases where perfusion increases due to the pruning of hypoperfused vessels, rather than blood being rerouted. These results indicate the importance of considering network composition when determining the optimal irradiation strategy. In the future, we aim to use our findings to identify tumours that are good candidates for perfusion enhancement and to improve the efficacy of combination therapies.

## Author summary

Dysregulated tumour vasculature often contains hypoperfused blood vessels which inhibit the delivery of blood-borne anticancer therapies. Radiotherapy, used to treat more than half of all cancer patients, causes DNA damage to vascular endothelial cells, preferentially impacting smaller vessels, leading to their death and vessel pruning. At the same time, experiments measuring changes in tumour perfusion post-irradiation produce varying outcomes and, therefore, the impact of irradiation-induced vessel pruning on network-scale perfusion remains unclear. In this study, we use recent (*in vivo*) imaging data to identify features of tumour vascular architectures that impact perfusion change post-irradiation. We then use a newly-developed computational framework, directly informed by the experimental observations, to elucidate the relationship between the vascular geometry and topology prior to radiotherapy and the irradiation-induced changes to network perfusion. We find that perfusion increases most significantly for networks of blood vessels with small mean diameters and large numbers of angiogenic sprouts. Our results also distinguish different mechanisms of perfusion increase and we identify cases where rerouting of blood flow causes previously hypoperfused vessels to become perfused. Our study sheds more light on the impact of radiotherapy on tumour blood flow; these insights could be useful for improving anti-cancer treatments.

## Introduction

Tumour cells exploit various biological opportunities to disregard the extracellular signals that normally direct an organism’s somatic cells to sacrifice themselves for the sake of its germ cells [1]. One of these opportunities is a tumour’s ability to incite the growth of its own blood supply network in a phenomenon called ‘tumour angiogenesis’. Tumour angiogenesis produces vasculature that grows in a disordered manner. Therefore, blood circulation in a tumour is sluggish, which leads to poor perfusion and a lack of oxygen. Poor oxygenation, in turn, attenuates the effect of major oncological therapies. The efficacy of radiotherapy, which is used to treat more than half of all cancer patients, depends on the generation of reactive oxygen species that induce irreparable DNA damage [2]. The mechanism governing the increased efficacy of radiotherapy in the presence of oxygen is explained by the ‘oxygen fixation hypothesis’ [3, 4]. Upon irradiation, free electrons are generated that mediate irradiation-induced damage, either by directly damaging the macromolecules, resulting in sites of DNA base loss and DNA single-strand breaks (SSBs), or by interacting with oxygen to form peroxyl radicals, which induce DNA damage, including double-strand breaks (DSBs) that are difficult for cells to repair [5, 6]. In hypoxia, the lower amount of oxygen results in reduced formation of reactive oxygen species (ROS), including peroxyl radicals. Hence, there is less DNA damage, leading to a greater ability for the damage to be repaired, resulting in increased radioresistance. Importantly, pockets of hypoxia within a tumour can exhibit a lower response to radiotherapy by up to a factor of three [7]. Moreover, adaptations to hypoxia can lead to tumour phenotypes with increased chemoresistance and metastatic capabilities [8]. For a list of drugs that perform more poorly in hypoxic conditions than in normoxic conditions, see [9].

Tumour growth is primarily monotonic, i.e., the tumours continue to grow and the tumour vasculature also grows continuously after the angiogenic switch has been triggered. Consequently, there is no spontaneous regression of the tumour blood vessels, resulting in a chaotic and disorganised vasculature with limited remodelling and maturation. In the development of normal vessels, one of the important mechanisms regulating their complexity and organisation is the trimming of a vascular network, termed ‘vessel pruning’, which marks the physiological regression of a subset of microvessels within a growing vasculature [10–12]. This phenomenon was studied in detail in the postnatal mouse retinal vascular network where it was shown that the vessel density at the sprouting front of a postnatal retina is significantly higher than in the mature vascular network of the adult retina. The main driver of vessel pruning is the vascular endothelial growth factor (VEGF) and VEGF receptor (VEGFR) signalling. However, other signalling pathways activated by fibroblast growth receptor 2 (FGF2), angiopoietin 2 (ANG2), platelet-derived growth factor (PDGF), and delta-like 4 (DLL4) have been shown to work independently of the VEGF/VEGFR pathway [11]. Anti-angiogenic therapies take advantage of these signalling axes and can cause a transient induction of vessel pruning in tumours, leading to a more normal-looking and stabilised vasculature. This is called ‘vascular normalisation’, which involves not only morphological but also functional improvements: reduced interstitial fluid pressure (IFP), reduced tumour hypoxia, and improved penetration of macromolecules from these vessels, which significantly improves the efficacy of some therapies, such as radiotherapy and chemotherapy [10, 13]. Recently it was shown that radiotherapy can also act as a vascular normalization therapy by preferentially pruning the small, non-perfused vessels [14, 15]. Importantly, non-perfused vessels and vessels with sluggish blood flow are also predisposed to vessel pruning and changes in blood flow act as regulators of vessel regression or maturation. In the mouse retina, a pharmacological inhibition of vasoconstriction or induction of vessel dilation resulted in reduced blood vessel stability confirming that vessel pruning is dependent on haemodynamic forces [16, 17]. However, it is not yet clear whether molecular components, such as angiogenic factors, or mechanical factors, such as shear stress, are the primary regulators of vessel regression associated with flow.

In the clinical setting, irradiation of the normal tissue surrounding the tumour cannot usually be avoided. In order to reduce the normal tissue toxicity of radiotherapy, the total radiation dose is divided into several fractions. ‘Conventional fractionation’ has been used in clinics for decades and divides the total radiation dose into 1.8–2 Gy fractions administered 5 days a week up to a total dose of 40–70 Gy. This type of fractionation takes advantage of the biological differences between the tumour and normal tissue, which usually results in less damage to the normal tissue with the same degree of tumour control [5]. In contrast to conventional low-dose fractionated therapies, hypofractionation describes a radiation treatment in which the total dose is administered in a smaller number of larger (>2 Gy) fractions, resulting in a shorter overall treatment time. If the treatment is administered as a single dose or in a small number of fractions, usually with a dose of 8–30 Gy per fraction, it is referred to as stereotactic body radiation therapy (SBRT) [18]. The use of SBRT has increased over the past decade. This is primarily due to technological advances in image guidance and highly conformal dose delivery, which allow a high dose of radiation to be delivered to the tumour while maintaining an acceptable dose to surrounding normal tissue [5, 18].

Experiments conducted to measure the change in tumour perfusion after irradiation of tumours have recorded varying outcomes. A review by Park et al. found that reported results were inconsistent, and that functionality improved and then worsened in human tumour vasculature during the early stages of radiotherapy [19]. Conversely, in human tumour xenografts or murine tumours, irradiation resulted in mild to severe damage depending on the dosage, reducing blood perfusion [19]. Kim et al. found that stereotactic body radiation therapy decreased perfusion [20]. On the other hand, Shibuya et al. found that in the case of cervical cancer, blood flow improved significantly after irradiation [21]. Moreover, a study by Bussink et al. found that irradiation led to rapid changes in perfusion, observing increases shortly after irradiation followed by significant decreases [22]. Kaeppler et al. used a transgenic CreERt2-tdTomato mouse model with <95% of endothelial cells fluorescently labelled coupled with *in vivo* intravital multiphoton microscopy which allowed them to simultaneously image the perfused and non-perfused tumor vasculature to study its response to single-dose and fractionated radiotherapy [15]. They used two tumour cell lines—a highly vascularised colon adenocarcinoma (MC38) and a less vascularised melanoma (B16F10)—and found that the former contained a larger proportion of hypoperfused vessels compared to the latter. Moreover, it was observed that the smaller-diameter vessels were more likely to be hypoperfused and also more likely to be pruned following irradiation (i.e., their endothelial cells were more susceptible to apoptosis). Thus, it was concluded that tumour perfusion post-irradiation depends on the density of small (typically hypoperfused) vessels.

An improved understanding of the relationship between irradiation and perfusion would allow for accurately-planned fractionated radiotherapy. However, it remains unclear why certain tumours exhibit increases versus decreases in perfusion after irradiation. The blood vessels that comprise tumour vasculature have heterogeneous architectural characteristics that present a complex system resistant to experimental analysis. Thus, there is a need for computational tools to guide and complement experimental research.

Broadly speaking, tumour vascular architecture has been modelled spatially with synthetic networks that reflect biological characteristics or, more recently, by digitising the geometry of real tumour vasculature. Regular forking networks, constructed by Bernabeu et al. using pathological dimensions gleaned from experimental data, are highly ordered because they are generated using a simple set of deterministic rules and the resulting geometry (vessel diameters, lengths, and arrangement of vessels into the network) is thus very regular [23]. Moreover, these networks exhibit strict hierarchy in that every parent vessel subdivides into two daughter vessels that are shorter and thinner than the parent. Non-hierarchical and ordered vasculature has been previously represented as a network with a repeated hexagonal unit, similar to that observed in avian yolk sacs [24]. Alarcón et al. have used a similar network to couple processes at the intracellular, cellular, and tissue scales [25]. Owen et al. employed both hexagonal and disordered networks to show that vascular remodelling could be achieved with a balance of pruning and angiogenesis [26].

Synthetic networks are also commonly generated by simulating the random migration of capillary tips based on a chemotactic gradient. Anderson and Chaplain developed a model using a random walk biased towards higher TAF (tumour angiogenesis factor) levels, which resembled *in vivo* angiogenic networks [27]. Similar models of biased random motility and sprout formation have been employed by Macklin et al. and Shirinifard et al. in 2D and 3D, respectively [28, 29]. In addition, Stepanova et al. have employed a multiscale random walk model to study endothelial cell dynamics [30]. The modelling of angiogenesis still remains an active area of research [31–33].

While synthetic networks can replicate many features of real tumour vasculature, some studies have gone a step further and digitised experimentally-acquired tumour vasculature. Grimes et al. used a 3D digitised network to estimate oxygen distribution [34]. Grogan et al. used a similar method to compare 2D and 3D representations, while Sweeney et al. used a 3D model to suggest that using realistic vasculature was key to modelling tumour fluid dynamics [35, 36]. However, such simulations presuppose the knowledge of boundary conditions and the methods for direct observation of flow in individual vessels are limited (especially smaller microvessels) [37].

Topological data analysis (TDA) is an emerging mathematical field that uses topological and geometric approaches to quantify the ‘shape’ of data [38, 39]. TDA characterises shape via topological invariants such as connected components and loops at multiple scales. The most prominent method from TDA, persistent homology, has been successfully applied to quantify dynamic characteristics of vascular networks in experimental data [40, 41] and to distinguish between synthetic vascular networks produced by different parameter regimes in a mathematical model of tumour-induced angiogenesis [42]. Persistent homology tracks topological features across a filtration, e.g., a nested sequence of vascular networks, and outputs information on the persistence of these features in the filtration in the form of a barcode [39]. The information contained in the barcode can either be vectorised and integrated with statistical or machine learning tools [43, 44] or it can be compressed into a single topological descriptor of the data, e.g., the number of loops across the filtration, as in [41]. In experimental data, the change in the total number of loops in vascular networks over time following radiotherapy has shown great variation across different tumours relative to the day of irradiation (single dose) [41]. However, this study did not take perfusion into account and, in particular, did not differentiate between different initial structural characteristics of the networks pre-therapy which can affect their response to radiotherapy. More recently, a novel topological descriptor for weighted directed graphs was developed [45]. For a weighted directed graph, e.g., a vascular network weighted by vessel diameter or flow time with the directions given by the flow, the method outputs a barcode describing its structure. While this topological descriptor encodes rich information about such a network, it has not yet been applied to real data, is computationally intensive, and would need to be substantially compressed to enable interpretable comparisons of multiple networks. Here, we focus on combining simple topological and geometrical information to describe the structure of vascular networks interpreted as undirected and unweighted graphs, i.e., disregarding information on flow direction and vessel diameters. We then link these structural features to changes in perfusion via flow simulations where flow direction and vessel diameters are taken into account. We are motivated by previous TDA studies of tumour vasculature [40–42], in which the number of loops appeared to reflect significant structural changes of vasculature. In contrast to these studies, we do not apply persistent homology, but focus on the total number of loops in the networks as a topological descriptor at consecutive time points. This topological descriptor is readily computable via the Euler characteristic (see the section **Topological determinants of perfusion**), does not require nestedness of the networks (in contrast to persistent homology), and is thus more suited to the analysis of networks undergoing pruning. None of the previous studies directly explored the link between network topology and perfusion as altered following irradiation and to the best of our knowledge, there exists no experimentally-motivated computational study of the effect of irradiation-induced pruning on network perfusion that considers both geometric and topological architectural features.

In this paper, we aim to characterise the extent to which radiotherapy-induced changes in vascular geometry (e.g., pruning of small vessels) may contribute to increased vascular perfusion. We identify key geometric and topological descriptors characterizing vasculatures with enhanced perfusion following vessel pruning as well as mechanisms by which such enhancement might occur. While [15] provided a broad analysis comparing two tumour cell lines under various radiotherapeutic treatment regimes, we noticed that even within one cell line (MC38) under single-dose radiotherapy, some tumours increased and some decreased their perfusion. We will therefore focus solely on MC38 under single-dose radiotherapy and aim to assess whether certain structural features pre-irradiation can be used as a proxy for predicting perfusion response to radiotherapy.

**Fig 1:**
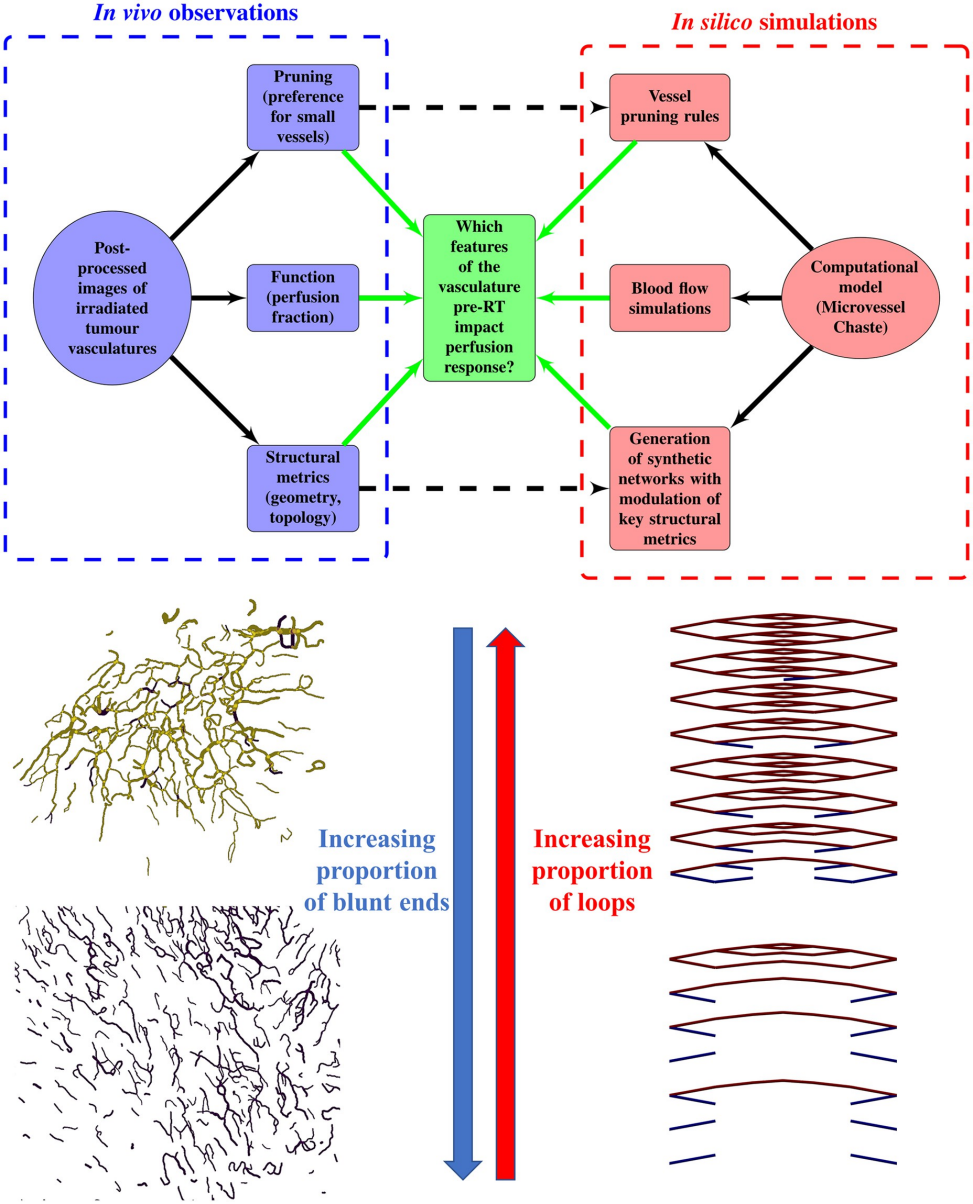
Chart summarizing the key components of the present study. Bottom-left panels present representative vascular regions containing perfused (yellow) and hypoperfused (purple) vessels from Day 0 (just before irradiation) for one of the tumours for which irradiation-induced vessel pruning led to a decrease in perfusion (tumour 6; top) and one for which it led to an increase (tumour 1; bottom). Note that the latter vasculature contained many hypoperfused blunt-ended vessels, while the former contained more loops. Bottom-right panels show pruned synthetic (forking) networks exhibiting similar properties with perfused (red) and hypoperfused (blue) vessels.

The structure of this work is summarised in Fig 1. Having introduced the biological and computational background for this study, we next describe the *in vivo* experimental study motivating and informing our model in the section **Experimental motivation**. In this section, we also highlight key correlations between certain architectural (geometric and topological) metrics and changes in perfusion after irradiation. In the section **Model overview**, we introduce key aspects of our computational model including the proposed architecture of the initial networks, the pruning rules, and the way in which perfusion is measured. We then use our model to investigate the underlying causal links and present two mechanisms of perfusion improvement in the **Results** section. We conclude with a contextualisation of our findings and a discussion of their implications in the **Discussion** section. The complete experimental procedure and model description can be found in the **Materials and Methods** section.

## Experimental motivation

### Uncertainty in direct flow simulations

Ideally, one would assess the impact of radiotherapy-induced vessel pruning on network perfusion by performing direct blood flow simulations on networks extracted from microscopy images. However, information about which network nodes serve as inlets and outlets is often limited or non-existent. Even given such information, it would still be challenging to determine whether constant pressure or constant flow rates should be imposed at inlets, and what values these pressures or flow rates should take to obtain quantitative agreement with measurements of blood flow *in vivo*. Despite receiving more attention from the scientific community in recent years, the problem of reliably assigning appropriate conditions at boundary nodes remains unresolved. In [37], an algorithm was developed whereby the boundary conditions are fitted so that the deviation of pressures and wall shear stresses in microvessels from appropriately selected target values are minimized. This method has been used in recent years to find appropriate boundary conditions for cerebral haemodynamics as well as in tumour perfusion simulations [46, 47]. However, due to the complexities of the dynamic tumour microenvironment, a reliable estimation of flow boundary conditions remains an active area of research. In this work, we choose a different strategy. First, in the section **Understanding perfusion response to radiotherapy and its determinants**, we identify key global geometric and topological metrics that can be used to determine whether irradiation-induced vessel pruning improves or impairs the perfusion of vascular networks extracted from tumours. Then, in the following sections, we present a theoretical study of network perfusion and vessel pruning in synthetic networks. In doing so, we aim to confirm the utility of the newly-proposed metrics in distinguishing tumour vascular networks whose perfusion increases or decreases following radiotherapy.

### Understanding perfusion response to radiotherapy and its determinants

Exposure to radiotherapy causes DNA damage in endothelial cells, which was found to induce cell death due to apoptosis preferentially in smaller hypoperfused vessels and result in cell cycle arrest in larger vessels which thus remain functional channels for the flow of blood [15, 48]. Regardless of the exact mechanism driving the cell death, we assume that radiation-induced DNA damage is the dominant cause of vessel pruning with a strong preference for small vessels and we neglect a preferential regression of hypoperfused vessels found in developmental vascular networks [17, 49].

We define perfused and hypoperfused vessels as in [15] (see also the section **Experimental procedures and data preprocessing** for the experimental procedure and the section **Perfusion threshold** for the minimum differentiating flow rate in our simulations) and similarly use the perfusion fraction (PF) to quantify network perfusion. The perfusion fraction P(t) is the ratio of the number of perfused vessels to the total number of blood vessels, i.e.:

$$\begin{array}{c} P\left(t\right)=\frac{\#\;\text{of}\;\text{perfused}\;\text{vessels}\;\text{at}\;\text{time}\;t}{\#\;\text{of}\;\text{remaining}\;\text{(unpruned)}\;\text{vessels}\;\text{at}\;\text{time}\;t}. \end{array}$$ (1)

**Fig 2:**
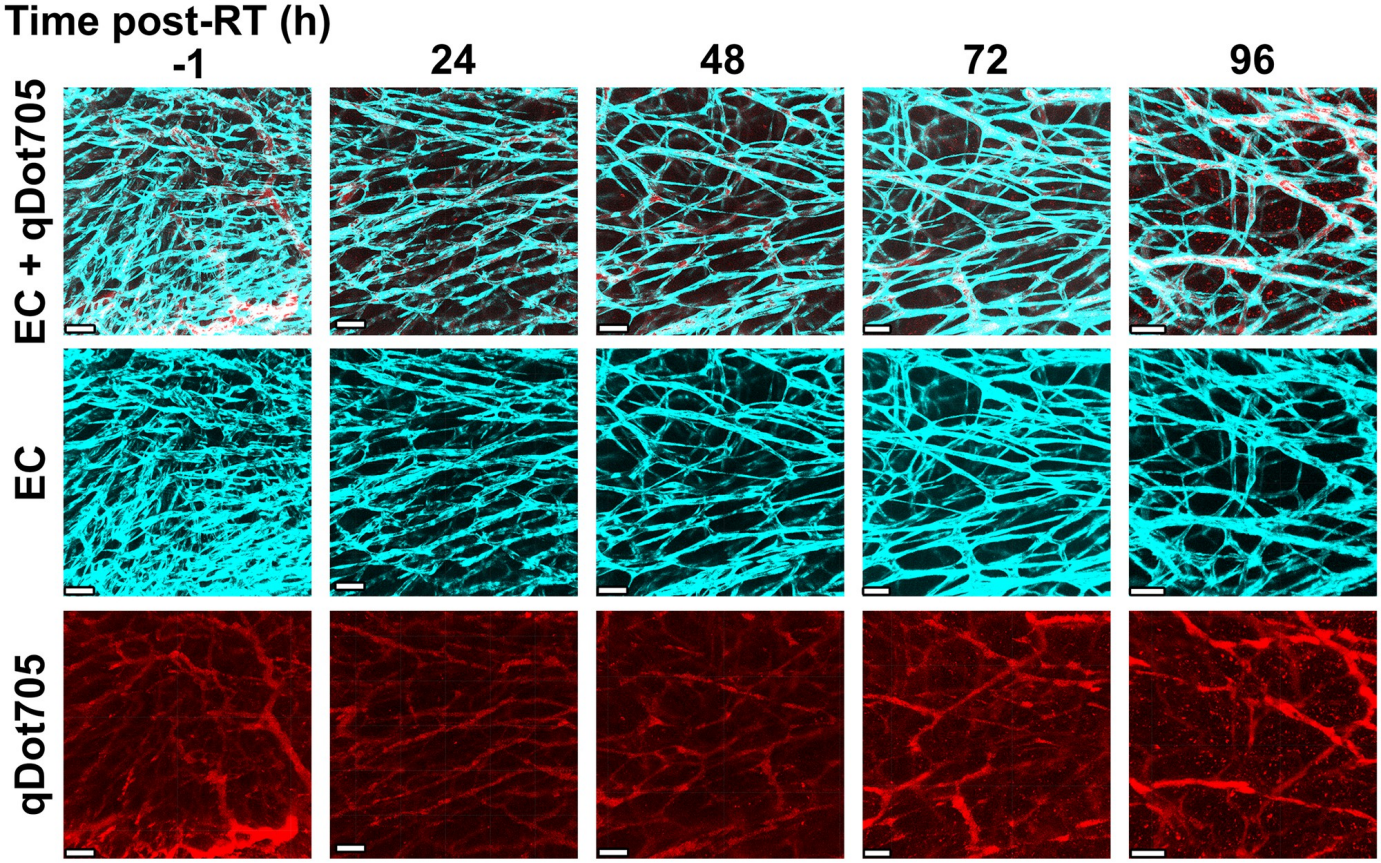
Representative vascular architectures from our experiments [15]. A single dose of 15 Gy X-rays was delivered to a tumour and changes to its vascular structure were monitored over the course of 4 days (reproduced from [50]). In the top row, endothelial cells are in cyan, while perfused vessels (qDot705) are in red. Every panel is a 2D representation of a 3D image in the form of a Z-stack approximately 300 μm tall. The depth of field for a single Z-slice was 2 μm. The middle and the bottom row show images of endothelial cells and perfused vessels, respectively. The scale bar corresponds to 100 μm.

**Fig 3:**
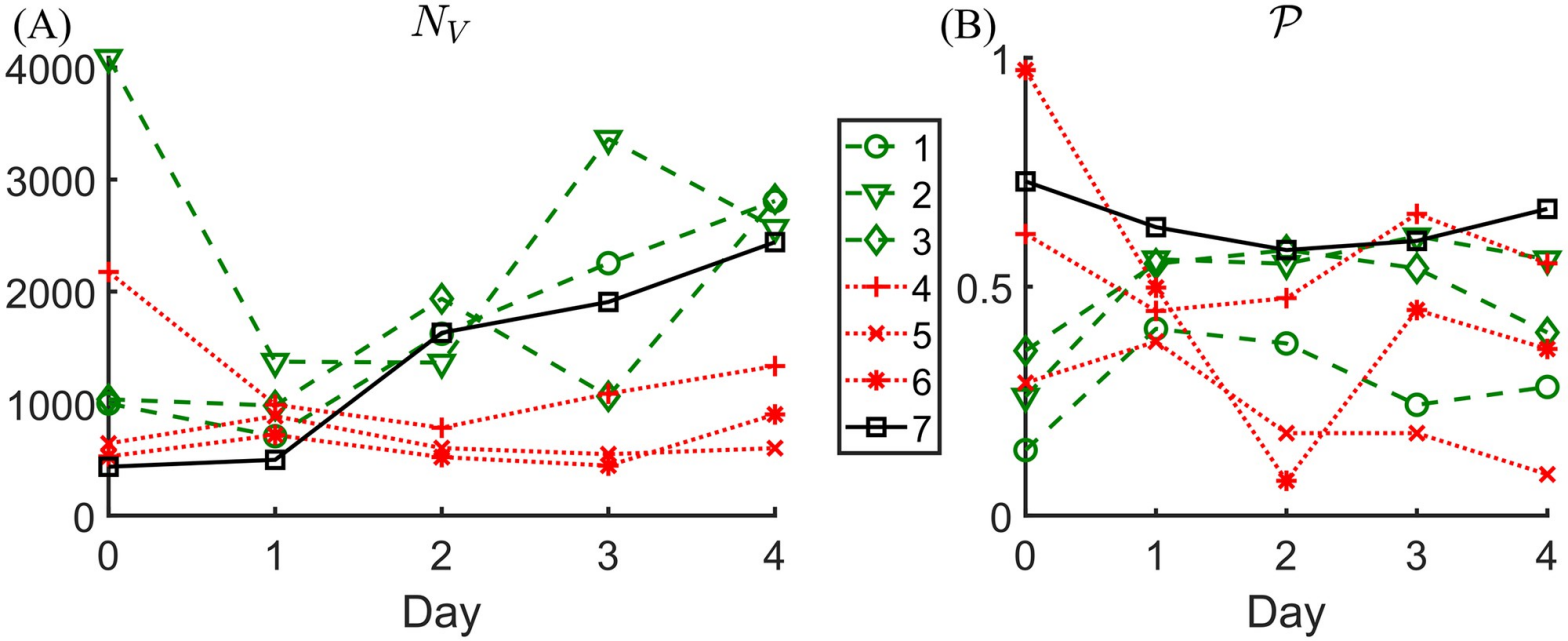
The first four days post radiotherapy (Day 0 is the day of irradiation). Time evolution of (A) vessel count *N*_*V*_ and (B) perfusion fraction P for the 7 tumours studied in [15].

The perfusion fraction is time-dependent because both the total number of vessels and the number of perfused vessels change over time, due to pruning (see the representative example in Fig 2). Experimental observations of irradiated tumours suggest that vessel pruning occurs on timescales that range from hours to days [15, 48, 51]. We thus focus on short-term responses, during the first four days following radiotherapy (Fig 3). We number the studied tumours from 1 to 7. Firstly, we note that the time between irradiation (Day 0 in Fig 3) and the first day on which a reduction in vessel number (vessel pruning) was observed varied across the tumours that were studied. While most tumours (1, 2, 3, and 4) exhibited a decrease in vessel number on Day 1, others (5 and 6) experienced a slight increase in vessel number over this time period. We note further that this delayed decrease in vessel number was observed for tumours with a relatively low vessel number and (at the same time) a large average vessel diameter at the time of radiotherapy (see Table 1). We speculate that these tumours were still undergoing extensive angiogenesis and had not developed a fully functioning tumour microvasculature at the time of radiotherapy.

**Table 1:** Key geometric and topological characteristics of tumour vasculatures on Day 0. Note that all lengths are in μm, all geometric resistances (total, mean, and per loop) in μm^−3^, and all resistances with viscosity in cP⋅μm^−3^, where cP (centipoise) is a unit of dynamic viscosity equal to mPa⋅s).

Change in PF ΔP	Increase (group A)			Visualization	Decrease (group B)		
Tumour number	1	2	3		4	5	6
Vessel count (size) *N*_*V*_	1000	4087	1038	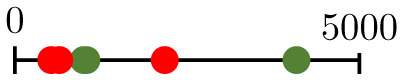	2175	644	528
Mean diameter $\bar{d}$	22.75	24.63	26.10	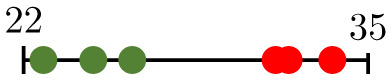	32.35	33.65	31.51
Mean length $\bar{L}$	112.70	106.48	121.47	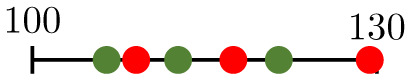	129.39	109.05	117.51
Total geometric resistance $R_{T}^{geom}$	0.95	3.79	0.80	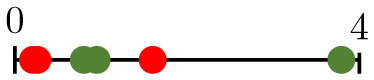	1.60	0.21	0.26
Total resistance with viscosity $R_{T}$	72.87	224.17	47.14	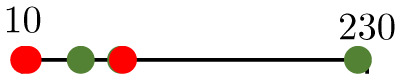	74.11	11.06	13.25
Mean geometric resistance ${\bar{R}}^{geom}$ (×10^−4^)	9.47	9.28	7.70	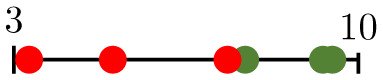	7.34	3.31	5.01
Mean resistance with viscosity $\bar{R}$ (×10^−2^)	7.29	5.48	4.54	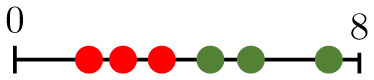	3.41	1.72	2.51
Loops *β*_1_	22	247	48	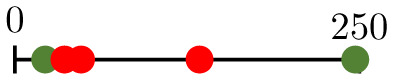	134	48	36
Loops per vessel $\bar{\beta_{1}}$ (×10^−2^)	2.20	6.04	4.62	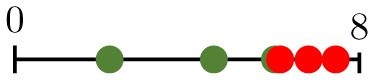	6.16	7.45	6.82
Resistance per loop ${\bar{R}}_{\beta }^{geom}$ (×10^−2^)	4.30	1.54	1.66	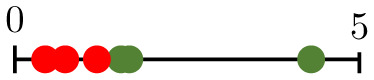	1.19	0.44	0.73
Resistance with viscosity per loop ${\bar{R}}_{\beta }$	3.31	0.91	0.98	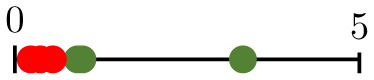	0.55	0.23	0.37

We note also that the vessel count for tumour 7 increased up to, and including, Day 4. This tumour’s vasculature had the largest number of connected components per size, and the size of its largest connected component was the smallest across all tumours (see Table A in S1 Appendix). These data are indicative of problems in image processing for this tumour and, therefore, we exclude it from our analysis. Secondly, the number of days over which the number of vessels decreased varied between tumours: for some tumours (1 and 3) the decrease only lasted one day, while for others (2, 4, 5, and 6) it lasted two days. This variability is likely due to the complex nature of the tumour microenvironment and uncertainties in the timescales for vessel pruning. The pruning phase was followed by a period characterised by a significant increase in vessel number, likely due to angiogenesis. Based on the above observations, we divide the tumours into two groups A and B so that a tumour belongs to group A or B if its perfusion fraction increases or decreases respectively during the pruning phase, which we define to be the period from Day 0 to the last day on which the number of vessels decreased, prior to the onset of angiogenesis. For a more quantitative comparison, we also define the pruning-induced perfusion difference ΔP and its relative counterpart $\Delta_{\%}P$ as follows:

$$\begin{array}{c} \Delta P=P\left(\text{Final}\;\text{Day}\right)-P\left(\text{Day}\;\text{0}\right)\;\text{and}\;\Delta_{\%}P=\frac{P(\text{Final}\;\text{Day})-P(\text{Day}\;\text{0})}{P(\text{Day}\;\text{0})}\times 100. \end{array}$$ (2)

The perfusion increases if $\Delta_{\%}P>0$ and decreases if $\Delta_{\%}P<0$. In Table 2, we summarize these quantities and rank the tumours with respect to the relative change in PF (as measured by $\Delta_{\%}P$).

**Table 2:** Perfusion fractions on Day 0 and the final day of the pruning phase, the pruning-induced perfusion difference, and its relative counterpart for six studied tumours. Tumours are ordered based on the relative change in PF as measured by $\Delta_{\%}P$.

Tumour	P(Day 0)	P(Final Day)	ΔP	$\Delta_{\%}P$
1	0.14	0.41	0.27	192.86
2	0.26	0.56	0.30	115.38
3	0.36	0.55	0.19	52.78
4	0.62	0.48	-0.14	-22.58
5	0.29	0.18	-0.11	-37.93
6	0.97	0.45	-0.52	-53.61

It might seem that the response to radiotherapy can be predicted by whether or not the tumour is initially highly-perfused. Tumours that are initially highly-perfused (e.g., 4 and 6) tend to have impaired perfusion post radiotherapy while those that are initially hypoperfused tend to have improved perfusion post radiotherapy (e.g., 1 and 2). However, even in our small dataset, we can find a counterexample: tumour 5 experienced a low perfusion fraction on Day 0, and an even lower value post-pruning. The particular value of the perfusion fraction (on any day of the experiment) is expected to be strongly dependent on the number, location, and strength of the inlet vessels which are often unknown. However, in the absence of such information and provided the inlet vessels are not pruned, it may still be possible to predict the perfusion response of tumour vasculature to radiotherapy (as measured by the relative change in the perfusion fraction $\Delta_{\%}P$) based on geometric and topological characteristics of the vasculature on Day 0 (i.e., just before the irradiation). Next, we carefully investigate the relationship between key characteristics of the tumour vasculature on Day 0 and the perfusion response.

#### Network size (vessel count)

In general, the resistance of a vascular network to flow increases with the number of vessels. Therefore, one might naively expect that pruning vessels in a large network should always increase its perfusion and, consequently, that irradiation should improve perfusion at a rate proportional to the number of vessels in the network. Most of our networks are consistent with this principle. However, the PF decreased following irradiation for tumour 4, even though it has a large network (see Table 1). We conclude that the vessel count alone is insufficient to predict perfusion response to radiotherapy.

#### Geometric determinants of perfusion

We now consider geometric determinants of flow resistance in individual vessels. One typically assumes that the flow rate *Q* through a cylindrical vessel, subject to a pressure drop Δ*p* across its length *L*, satisfies Poiseuille’s law:

$$\begin{array}{c} Q=\frac{\Delta p}{R}\;\text{and}\;R=\frac{128L\mu^{\text{eff}}}{\pi d^{4}}, \end{array}$$ (3)

where *d* denotes vessel diameter and *μ*^eff^ is the effective viscosity of blood [23, 52]. This parameter depends in a nonlinear way on the vessel diameter and haematocrit (see [53]). Determining the haematocrit distribution within a network is challenging due to uncertainty in the locations and strengths of the inlets, the lack of consensus about the functional form describing haematocrit splitting (this remains an active research area [23, 54–56]), and the highly coupled and nonlinear nature of the haematocrit and blood flow. Therefore, for the sake of computational efficiency, we impose a uniform haematocrit *H* = 0.45 in all networks (the effect of varying *H* is documented in Section S1.5 of S1 Appendix). Substituting this value into standard formulas obtained in [53] (see Eqs (17)–(19)), we arrive at a relationship describing how the effective blood viscosity depends on vessel diameter (also known as the Fåhræus–Lindqvist effect) [57, 58]. Note that for consistency with existing mathematical models [53], all length scales in Eqs (17)–(19) are nondimensionalised with respect to 1 μm. Substituting this relationship into Eq (3), we obtain an explicit expression for the resistance R in a vessel of length *L* and diameter *d*. As a further simplification, we propose the quotient $R^{geom}=L/d^{4}$ as the simplest proxy for vessel resistance involving only the key geometric parameters. At the network scale, we define the following measures for the total resistance of a network:

$$\begin{array}{c} R_{T}=\frac{128}{\pi }\sum_{i}\frac{L_{i}\mu_{i}^{\text{eff}}}{d_{i}^{4}}\;\text{and}\;R_{T}^{geom}=\sum_{i}\frac{L_{i}}{d_{i}^{4}}, \end{array}$$ (4)

where the sum is over all network vessels *i*. Finally, we define the following proxies for mean vessel resistance:

$$\begin{array}{c} \bar{R}=\frac{R_{T}}{N_{V}}\;\text{and}\;{\bar{R}}^{geom}=\frac{R_{T}^{geom}}{N_{V}}. \end{array}$$ (5)

Due to the fourth-power dependence of the resistance on diameter, we expect that the average vessel diameter in a network will strongly impact its resistance. Table 1 confirms this: networks with small mean diameters typically have high mean resistance (both $\bar{R}$ and ${\bar{R}}^{geom}$). Furthermore, the mean diameter and mean resistances appear to be good predictors of radiotherapy response: low values of mean diameter (high values of mean resistance) correspond to group A, and vice versa. We note that neither the mean length nor the total resistance (which neglects network size, and decreases when vessels are pruned) distinguish between the two groups of tumours. In summary, for the 6 studied tumours, the mean diameter $\bar{d}$ and mean resistance $\bar{R}$ distinguish between the two groups, with group A having, on average, thinner (higher resistance) vessels than group B.

#### Topological determinants of perfusion

We use Betti curves [59, 60] to track the number of loops in vascular networks during radiotherapy-induced vessel pruning. The Betti numbers *β*_0_ and *β*_1_ refer to the number of connected components and the number of loops in a network, respectively. Via the Euler-Poincaré formula [60, 61], the Euler characteristic X of a network, which is given by its number of edges (vessels) *N*_*V*_ and nodes *N*_*N*_, i.e., $X=N_{N}-N_{V}$, is directly connected to its Betti numbers: $X=\beta_{0}-\beta_{1}$. Given *β*_0_, *N*_*V*_, and *N*_*N*_, *β*_1_ can therefore be computed as [60]:

$$\begin{array}{c} \beta_{1}=\beta_{0}-N_{N}+N_{V}. \end{array}$$ (6)

To obtain a Betti curve for *β*_1_, we track the number of loops throughout the pruning process on a network.

As in the previous section, we also introduce metrics normalised by the number of vessels, i.e.:

$$\begin{array}{c} \bar{\beta_{0}}=\frac{\beta_{0}}{N_{V}}\;\;\text{and}\;\;\bar{\beta_{1}}=\frac{\beta_{1}}{N_{V}}. \end{array}$$ (7)

We note from Tables 1 and A (the latter in S1 Appendix.) that *β*_0_, $\bar{\beta_{0}}$, and *β*_1_ do not distinguish between the two groups of tumours (A and B), whereas $\bar{\beta_{1}}$—which we will call loops per vessel—provides a (weak) distinction between the two groups: tumours with fewer loops per vessel belong to group A. To elucidate why the loops per vessel on Day 0 might impact the perfusion response, we use Eqs (6) and (7) to write:

$$\begin{array}{c} \bar{\beta_{1}}=\bar{\beta_{0}}-\frac{N_{N}}{N_{V}}+1. \end{array}$$ (8)

In what follows, we consider a network containing *N*_*I*/*O*/*ST*_ nodes of degree 1 (inlets, outlets, and tips of angiogenic sprouts that have not yet anastomosed) and *N*_*B*_ nodes of degree 3 (bifurcation points). For simplicity, we ignore nodes of degree 2 (vessels subdivided into segments), 4 (trifurcations), and higher as these seldom appear in our networks. We then have *N*_*N*_ = *N*_*I*/*O*/*ST*_ + *N*_*B*_ and, counting vessels twice by looping over all network nodes, we have *N*_*V*_ = (*N*_*I*/*O*/*ST*_ + 3*N*_*B*_)/2. Using these relations, Eq (8) can be simplified to read:

$$\begin{array}{c} \bar{\beta_{1}}=\frac{1}{3}+\bar{\beta_{0}}-\frac{2}{3}\frac{N_{I/O/ST}}{N_{V}}=\frac{1}{3}+\bar{\beta_{0}}-\frac{2}{3}\bar{N_{I/O/ST}}, \end{array}$$ (9)

where $\bar{N_{I/O/ST}}$ denotes the normalised number of degree-1 nodes. We see that small values of $\bar{\beta_{1}}$ result from large numbers of nodes of degree 1, and vice versa. As the degree of a node relates to its connectivity, vessels adjacent to degree-1 nodes are unlikely to play a significant role in network perfusion and pruning such vessels is desirable. Moreover, while some of these are inlet or outlet nodes, the majority are likely to represent tips of angiogenic sprouts (blunt ends) that have not yet anastomosed. Consequently, the relevant vessels are hypoperfused. Pruning these sprouts increases the perfusion fraction simply by reducing the denominator in Eq (1), whereas pruning vessels that connect bifurcation points (degree = 3) may disconnect parts of the network that were previously connected. This is confirmed in the bottom-left panels in Fig 1: the vasculature for which the PF increased (tumour 1; bottom) contains more angiogenic sprouts (i.e., is less inter-connected, with lower $\bar{\beta_{1}}$) on Day 0, than the one for which the PF decreased (tumour 6; top). Taken together, these results provide a possible explanation for why tumours with low $\bar{\beta_{1}}$ increase their PF, and vice versa.

#### Metrics combining geometry and topology

A full understanding of the determinants of network perfusion is still lacking. It requires more detailed knowledge of the diameter and length distributions within a network and their connectivity. The intricate (nonlinear) nature of blood rheology further complicates the situation. A single measure (geometric or topological) is unlikely to contain complete information about the perfusion fraction. To assess the impact of radiotherapy on the perfusion fraction, one needs to consider the distribution of high- and low-resistance loops that are being pruned from the network. To this end, and inspired by the observations above, we now propose new metrics that combine network topology with its geometric properties in the form:

$$\begin{array}{c} {\bar{R}}_{\beta }=\frac{\;\bar{R}\;}{\;\bar{\beta_{1}}\;}\;\text{and}\;{\bar{R}}_{\beta }^{geom}=\frac{{\bar{R}}^{geom}}{\bar{\beta_{1}}}. \end{array}$$ (10)

Table 1 shows that these metrics can distinguish between the two groups of tumours (particularly ${\bar{R}}_{\beta }$, which accounts for the dependence of viscosity on the vessel diameter). To our knowledge, these are the first global metrics to combine the geometry and topology of vascular networks.

Taken together, our analysis suggests that networks containing large numbers of high-resistance vessels and angiogenic sprouts are likely to increase their PF following radiotherapy. In other words, we postulate that irradiation-induced vessel pruning will increase the PF of vascular networks containing large proportions of thin and blunt-ended vessels—we will next test this hypothesis using synthetic networks.

## Model overview

**Fig 4:**
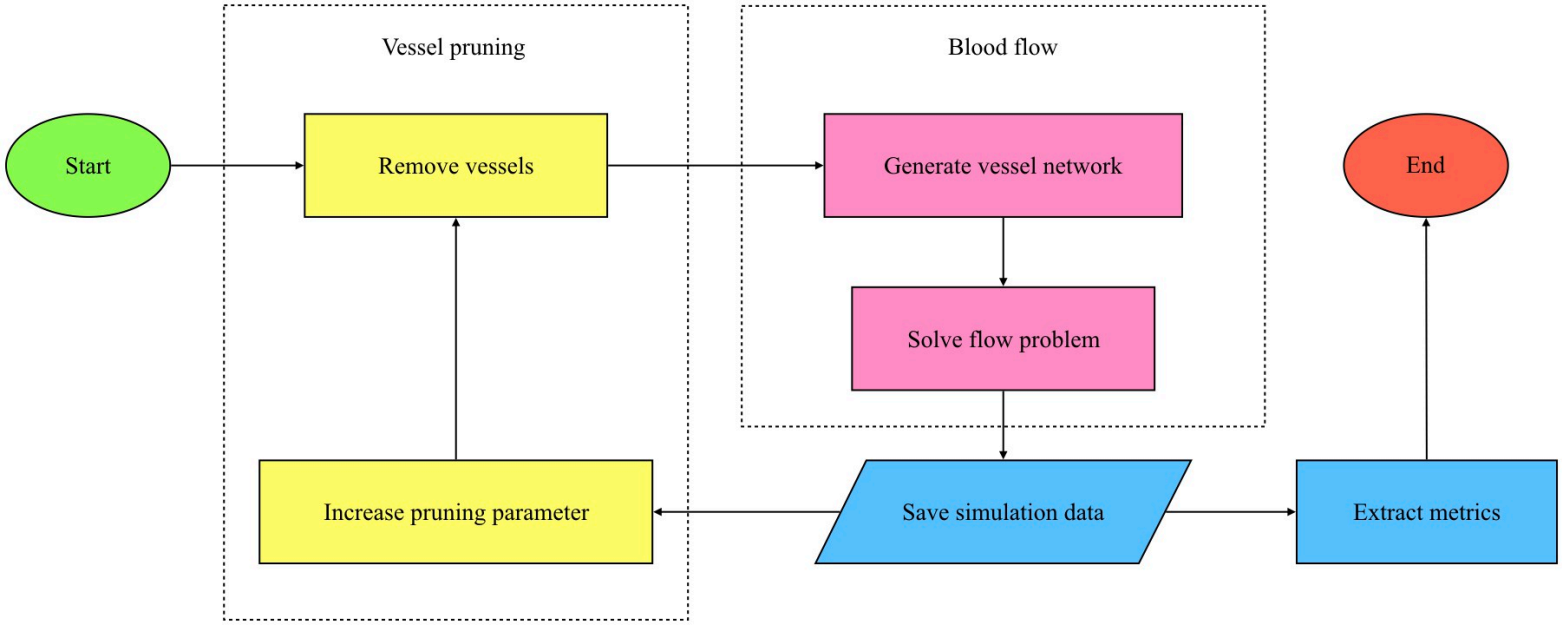
Flow chart summarising our model components. Simulations were carried out using Microvessel Chaste [62, 63].

Our analysis of the experiments revealed that vascular architecture influenced the outcome of pruning: tumours with lower mean vessel diameters ($\bar{d}$) and fewer vascular loops per vessel ($\bar{\beta_{1}}$) exhibited an increase in perfusion ($\Delta_{\%}P>0$) when irradiated. We then used our computational model to better understand the causal relationship between the architectural properties of a vascular network and how its perfusion changes following radiotherapy (Fig 4). In brief, we constructed multiple networks to replicate the characteristics of interest ($\bar{d}$ and $\bar{\beta_{1}}$) exhibited by the biological dataset. We then simulated blood flow through the networks and measured the change in perfusion as vessels were successively pruned. The design of the model is detailed below, with the parameters in Section S1.8 of S1 Appendix.

### Network design

We used forking and hexagonal networks to represent hierarchical and non-hierarchical vasculature respectively (Table 3). We then simulated blood flow through the networks. Using simple symmetric geometries allows us to isolate the influence of the different geometric and topological factors (e.g., vessel diameters, lengths, and network loops) under consideration. While the geometries of the networks are simple, they reflect several properties of biological vasculature. Here, we describe the forking networks since they form the focus of our results. The design of the hexagonal network can be found in Section S1.3 of S1 Appendix. In the forking network, each vessel divides into two daughter vessels until 7 generations are created (counting the inlet vessel as generation 0). After the seventh generation (generation 6), the network converges symmetrically into a single outlet vessel. The effect of varying the number of generations is documented in Section S1.6 of S1 Appendix. Note that throughout this work, for simplicity, we consider any straight-line segment to be an individual vessel (as opposed to defining vessels by their two endpoints being bifurcation nodes, inlets, outlets, or blunt ends).

**Table 3:** Heterogeneity in both hierarchical and non-hierarchical networks is modulated by a single parameter.

Architecture	Hierarchy	Heterogeneity Parameter	Values
forking	present	relative thickness of daughter vessels (*α*)	1.1, 1.2, 1.3
hexagonal	absent	SD of diameter distribution (*σ*)	8.68 μm, 13.23 μm, 17.49 μm

#### Vessel dimensions

The diameters of the two daughter vessels (*d*_*A*_ and *d*_*B*_) are related to the diameter of the parent vessel (*d_parent_*) via Murray’s law [64]:

$$\begin{array}{c} d_{parent}^{3}=d_{A}^{3}+d_{B}^{3}. \end{array}$$ (11)

Therefore, the inlet vessel diameter *d_inlet_* modulates the thickness of all subsequent vessels. Following [23], we assume that the vessel lengths *L* are proportional to vessel diameters (*d*) so that:

$$\begin{array}{c} L=\lambda d. \end{array}$$ (12)

Based on the mean vessel length to mean diameter ratios in our experimental data, we fix λ = 4. The branching angle at a bifurcation is dictated by the *y*-extent of its constituent vessels. The *y*-extent of a vessel in generation *i* ≥ 1 is the length of its projection onto the *y*-axis (*V*_i_) in a manner that places the following limit on the spatial extent of the network [23]:

$$\begin{array}{c} V_{i+1}=\frac{V_{i}}{2}. \end{array}$$ (13)

**Fig 5:**
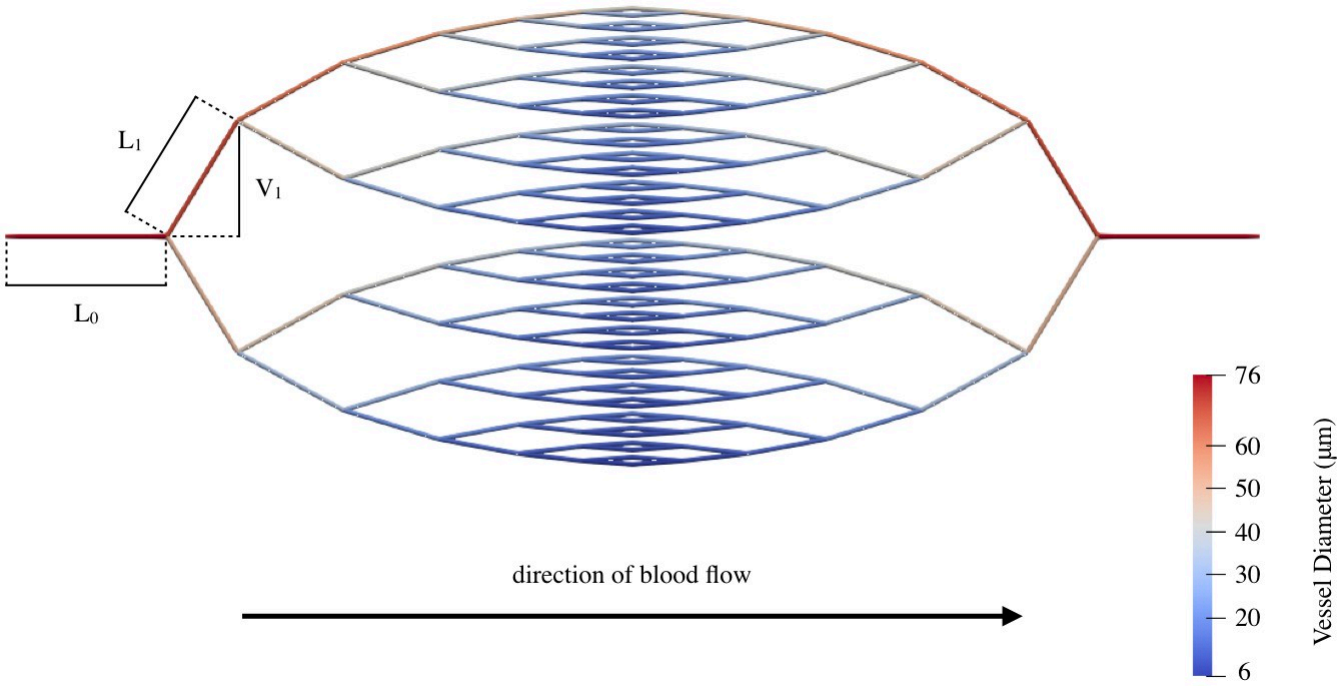
Blood flows from left to right through a single inlet and a single outlet in the forking network. *L*_i_ and *V*_i_ denote the length and *y*-extent of a vessel in generation *i*, respectively. Note that our implementation of network heterogeneity results in vessels at the top of the network being the thickest in their generation and vessels at the bottom being the thinnest.

We set *V*_1_ = 0.9*L*_1_ for our simulations to allow the network to extend to a sufficient degree along the *y*-axis, in line with [23]. If we further impose that two daughter vessels of any parent vessel must have the same diameter (*d*_*A*_ = *d*_*B*_), then the network geometry is fully specified. This forms our reference network (Fig 5). Our reference network must be modified to reflect the properties of interest ($\bar{d}$ and $\bar{\beta_{1}}$) in the biological networks. Therefore, we offset the mean vessel diameter of the synthetic networks to match the minimum, maximum, and average values of $\bar{d}$ observed across the biological vasculatures (see Section S1.2 of S1 Appendix for details on how we did so). Throughout our diameter variations, all vessel lengths and branching angles remain identical to the reference network. Therefore, Eq (12) does not apply in these cases. Variations in $\bar{d}$ do not result in variations in $\bar{\beta_{1}}$ within a network. Therefore, we tracked $\bar{\beta_{1}}$ as we pruned the forking network and measured the corresponding changes in perfusion. In effect, we considered every vessel removal as a way of generating a new network with a different $\bar{\beta_{1}}$.

#### Network heterogeneity

To ensure heterogeneous diameters in the network, we fixed the diameter of one of the two daughter vessels (*d*_A_) at each bifurcation to be *α*-times (*α* > 1) thicker than the other (*d*_B_):

$$\begin{array}{c} d_{A}=\alpha d_{B}. \end{array}$$ (14)

In the homogeneous case (*α* = 1.0), both daughter vessels have the same diameter. The diameter of a vessel in any generation can be calculated as:

$$\begin{array}{c} d_{i}=\frac{d_{inlet}\alpha^{n_{thick}}}{{\left(1+\alpha^{3}\right)}^{\frac{i}{3}}}, \end{array}$$ (15)

where *d*_*i*_ is the diameter of a vessel in generation *i* on a path that features *n*_*thick*_ thick vessels originating from an inlet vessel with diameter *d*_*inlet*_. Since every permutation of thick and thin vessels on a path exists in the network, it makes no difference which daughter vessel is *α*-times thicker than the other at each bifurcation. We choose the vessel that extends upwards from a bifurcation to be thicker than the lower vessel. As a result, vessels at the top of the network are the thickest in their generation and those at the bottom are the thinnest (Fig 5).

### Pruning

**Fig 6:**
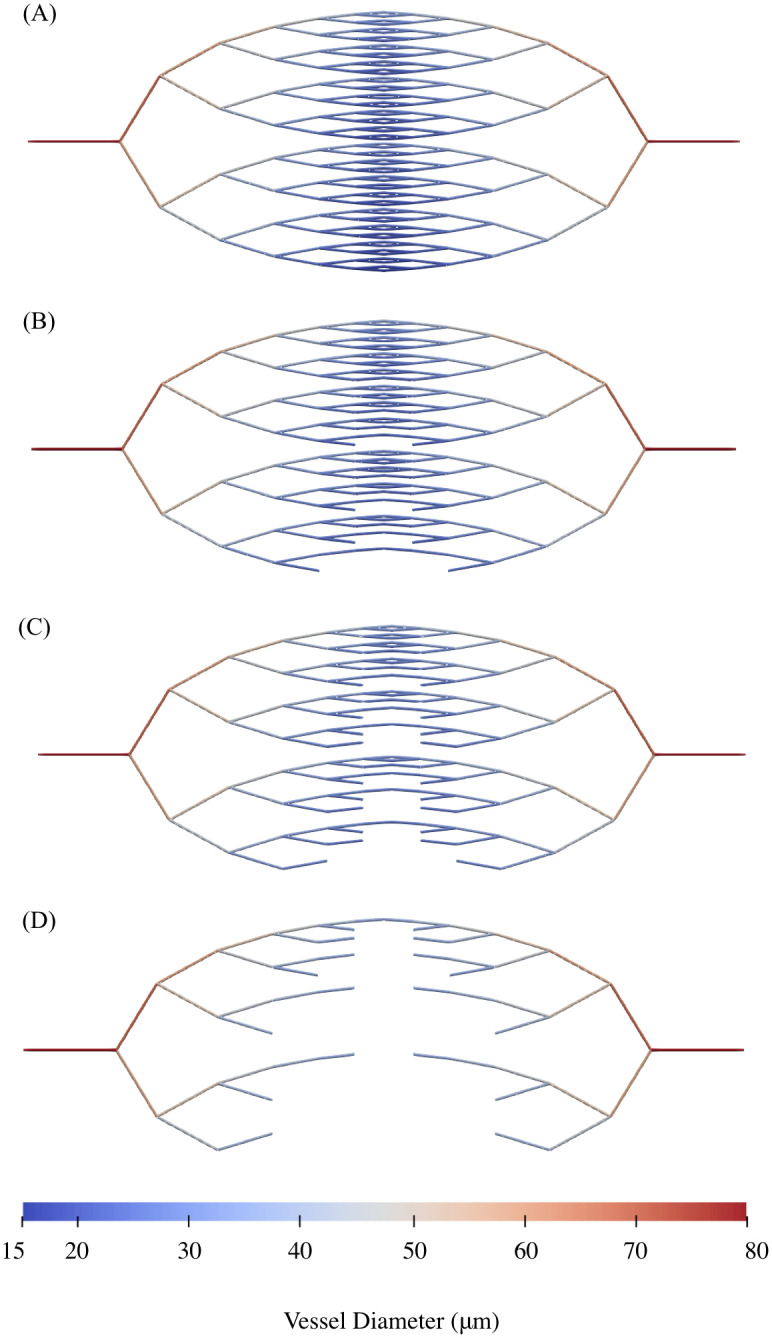
An example of pruning. Changes to the architecture of a forking network as (A) 0 vessels, (B) 50 vessels, (C) 100 vessels, and (D) 200 vessels are pruned.

Radiotherapy preferentially prunes smaller vessels and the extent of pruning increases with the dose [14, 15]. Therefore, we simulated increasing magnitudes of dosage by removing vessels individually in order of increasing diameter (Fig 6). Recall that the constant λ dictates the ratio between vessel lengths and diameters in the forking network. Therefore, pruning by diameter effectively prunes by length as well, but provides more data points than the 7 distinct lengths present in the forking network. Removing one vessel at a time allows us to examine pruning at a greater temporal resolution than obtainable experimentally. If two vessels have the same diameter then the vessels are pruned in order of their Vessel ID as detailed in Section S1.2 of S1 Appendix. The effect of pruning multiple vessels of equal diameters simultaneously is documented in Section S1.7 of S1 Appendix. Due to the lack of hierarchy, isolated vessels may remain in the hexagonal network during the course of pruning. Note that our model can only be used to mimic the effect of single-dose radiotherapy over relatively short time scales, as a reliable model of a fractionated radiotherapy over the course of multiple weeks would require one to take into account significant angiogenic growth and vascular remodelling.

### Perfusion threshold

Instead of identifying vessels as perfused based on fluorescence, we track the flow directly and introduce the perfusion threshold (*Q*_*min*_) as the minimum flow rate at, or above, which vessels are considered perfused and below which vessels are considered ‘hypoperfused’. This threshold serves as a proxy for the sensitivity with which our experimental apparatus can detect the signal coming from perfused vessels. In the forking network, we set the value of this threshold *Q*_*min*_ to be 3 × 10^−12^ m^3^ s^−1^ which, for the default model parameters and the three offsetting scenarios of the least heterogeneous network (*α* = 1.0), yields initial PFs of 0.24 (minimum), 0.50 (average), and 1.00 (maximum). In this way, we cover a large range of initial PFs spanning almost the entire interval (0, 1) which was also observed in real vasculatures (initial PFs ranging from 0.14 to 0.97). Similarly, we set *Q*_*min*_ = 3 × 10^−13^ m^3^ s^−1^ for the hexagonal network. This value allows us to cover a sufficiently large range of initial PFs.

### Perfusion fraction

We compute the PF using the experimental formula (Eq (1)). Guided by Eq (2), we quantify improvements in perfusion for synthetic networks by introducing $\Delta^{\text{max}}P$, the maximum value attained by ΔP during pruning, and $\Delta_{\%}^{\text{max}}P$, the maximum percentage change in the PF, where:

$$\begin{array}{c} \Delta^{\text{max}}P=P^{\text{max}}-P_{0}\;\text{and}\;\Delta_{\%}^{\text{max}}P=\frac{P^{\text{max}}-P_{0}}{P_{0}}\times 100. \end{array}$$ (16)

In Eq (16), $P_{0}$ and $P^{\text{max}}$ denote, respectively, the initial and maximum value of the PF during pruning.

## Results

In line with our analysis of actual tumours, we found that the largest increases in PF in our synthetic vascular networks were associated with lower values of $\bar{d}$ and $\bar{\beta_{1}}$. Additionally, we identified two mechanisms that can effect a positive change in perfusion post-irradiation. The vascular remodelling that occurs *in vivo* makes it difficult to isolate the effects of each mechanism. By contrast, synthetic networks offer no such barrier to analysis. We detail our inferences from these simulated networks in the following subsections.

While our forking networks are inherently hierarchical, the same (high) level of order might not be present in dysregulated tumour vasculatures. In order to gain insight into the role that hierarchy plays, we also examined the effect of non-hierarchical structures modelled as hexagonal networks and the impact of diameter heterogeneity. We found that hierarchy was conducive to perfusion enhancement and that heterogeneity resulted in an increased and sustained $\Delta_{\%}P$ response. For a full discussion, see Section S1.4 of S1 Appendix.

### Two mechanisms can increase the perfusion fraction post-irradiation

**Fig 7:**
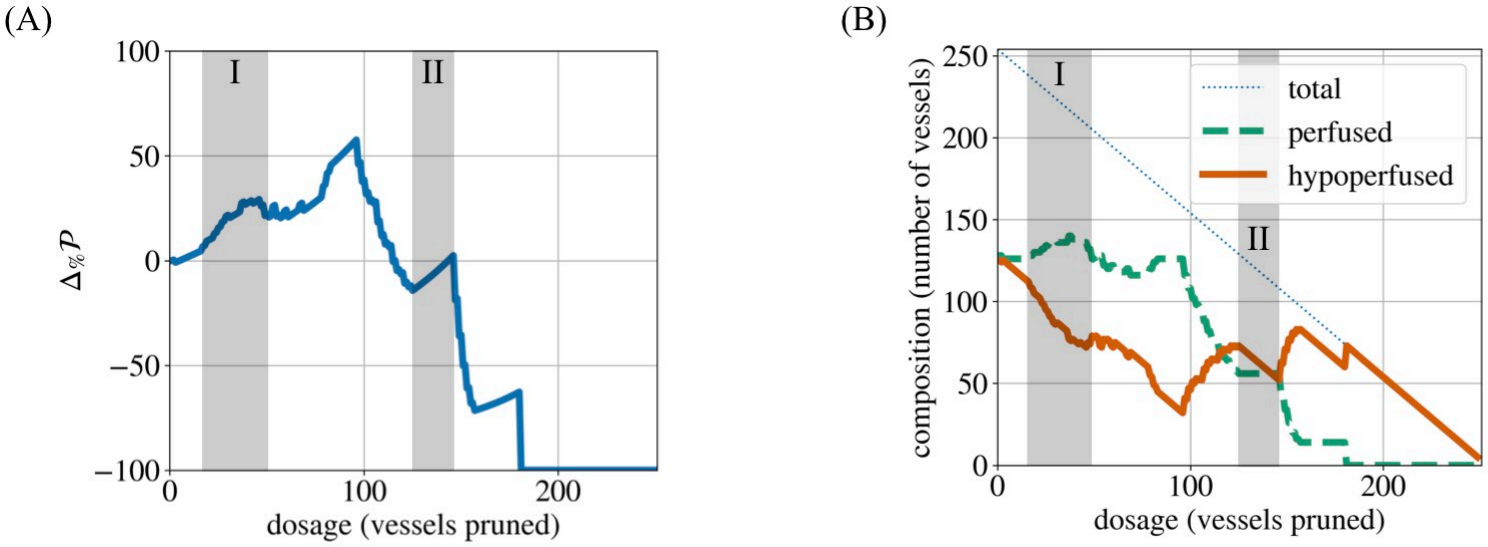
The two mechanisms of P increase. When a forking network (*α* = 1.1, $\bar{d}=28.50$ μm) is pruned, perfusion ($\Delta_{\%}P$) can increase, shown in (A), via Mechanism I when blood flow is rerouted from pruned vessels to other vessels, as seen in (B) where the number of perfused vessels increases and the number of hypoperfused vessels decreases. In contrast, perfusion ($\Delta_{\%}P$) can also increase, shown in (A), via Mechanism II when hypoperfused vessels are pruned without any net increase in the number of perfused vessels, as evidenced in (B) by the constant number of perfused vessels.

**Fig 8:**
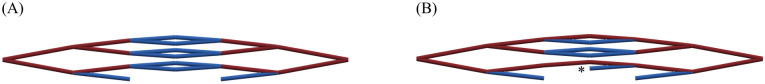
Flow rerouting. Flow is rerouted in this section of a forking network (*α* = 1.1, $\bar{d}=28.50$ μm) when a single vessel is pruned (original position marked with an asterisk) between (A) and (B) and the flow rate increases in four hypoperfused vessels (blue) to the extent that they become perfused (red).

One can deduce from Eq (1) that the perfusion fraction (P) can increase not only if the number of perfused vessels increases after pruning, but also if the number of hypoperfused vessels decreases (Fig 7). We conclude that two mechanisms can increase the value of $\Delta_{\%}P$ when a hypoperfused vessel is pruned:

- **Mechanism 1**: When a vessel is pruned, blood flow may be rerouted causing one, or more, hypoperfused vessels to become perfused and increasing the number of perfused vessels (Fig 8).
- **Mechanism 2**: Removing hypoperfused vessels increases the proportion, but not the number, of perfused vessels.

**Fig 9:**
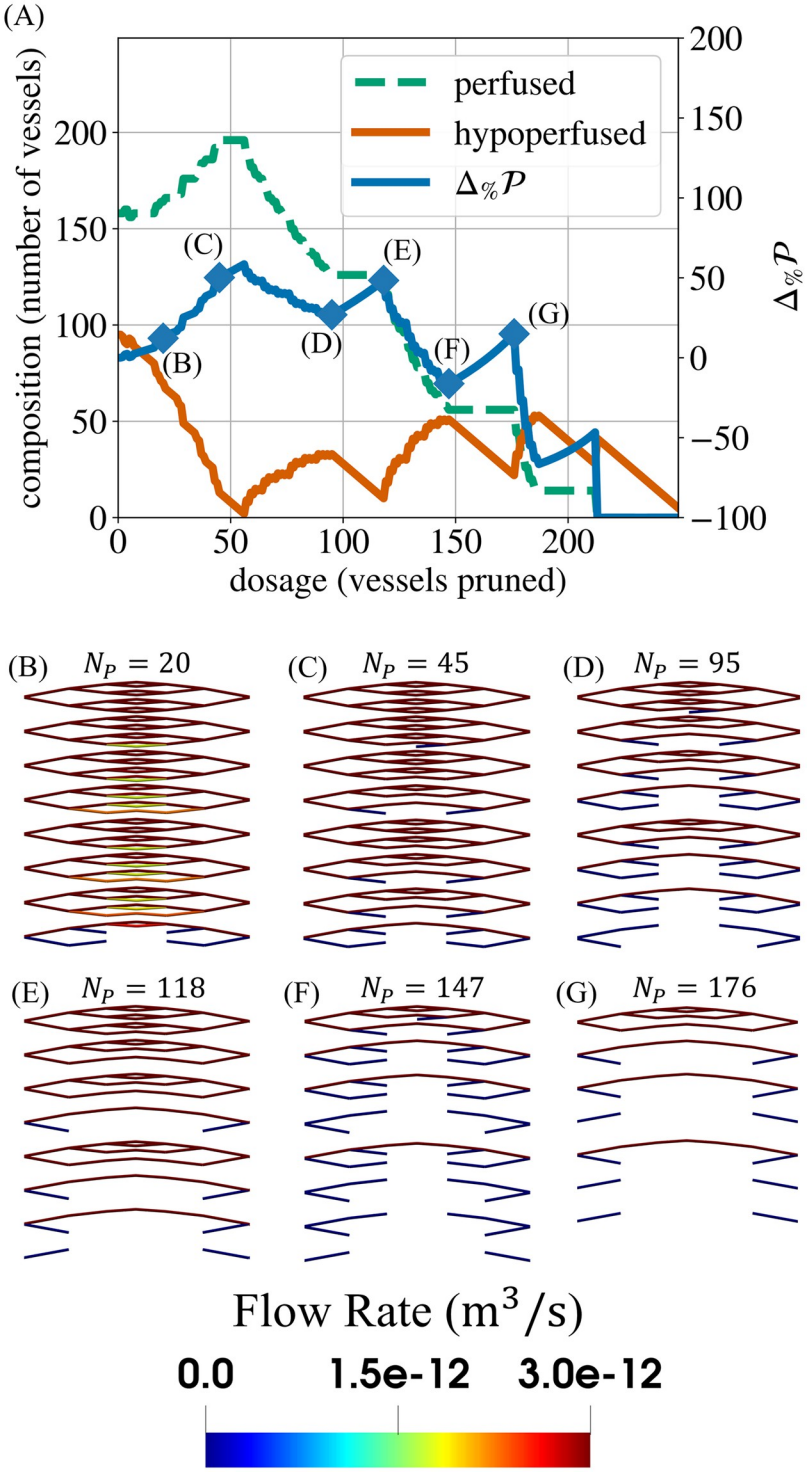
Analysing perfusion response. Panel (A) shows the perfusion response of a single forking network (*α* = 1.2, $\bar{d}=33.65$ *μ*m). Considering the central portion of this network, panels (B)–(G) present the progression of flow distributions in the process of pruning, noting that the maximum of the colourmap is set equal to the perfusion threshold (i.e., vessels that are not dark red are hypoperfused). Having pruned (B) the 20 thinnest vessels, the network contains pairs of daughter vessels which experience flow rates slightly below the perfusion threshold (*Q*_*min*_ = 3 × 10^−12^m^3^s^−1^). Pruning one vessel of such a pair is likely to result not only in (C) fewer hypoperfused vessels but also in more perfused vessels due to flow rerouting. Between (D) and (E) and between (F) and (G), we primarily prune blunt ends which increases the perfusion fraction ($\Delta_{\%}P$) via Mechanism 2. Between (E) and (F), $\Delta_{\%}P$ dramatically decreases because perfused paths are disrupted.

Fig 9 shows how both mechanisms act on a network during pruning. In the original, unpruned network, several hypoperfused vessels have blood flow rates that are close to the perfusion threshold while others have flow rates that are well below the perfusion threshold. When the least-perfused vessels are pruned, their flow is rerouted and the flow rate in the remaining vessels (several of which were previously hypoperfused) increases above the perfusion threshold. A decrease in the number of hypoperfused vessels alone would correspond to a network that becomes more efficient following radiotherapy. However, it would not necessarily correspond to an improvement in oxygen distribution.

### Mean vascular diameter determines the relative contribution of the two perfusion improvement mechanisms

**Fig 10:**
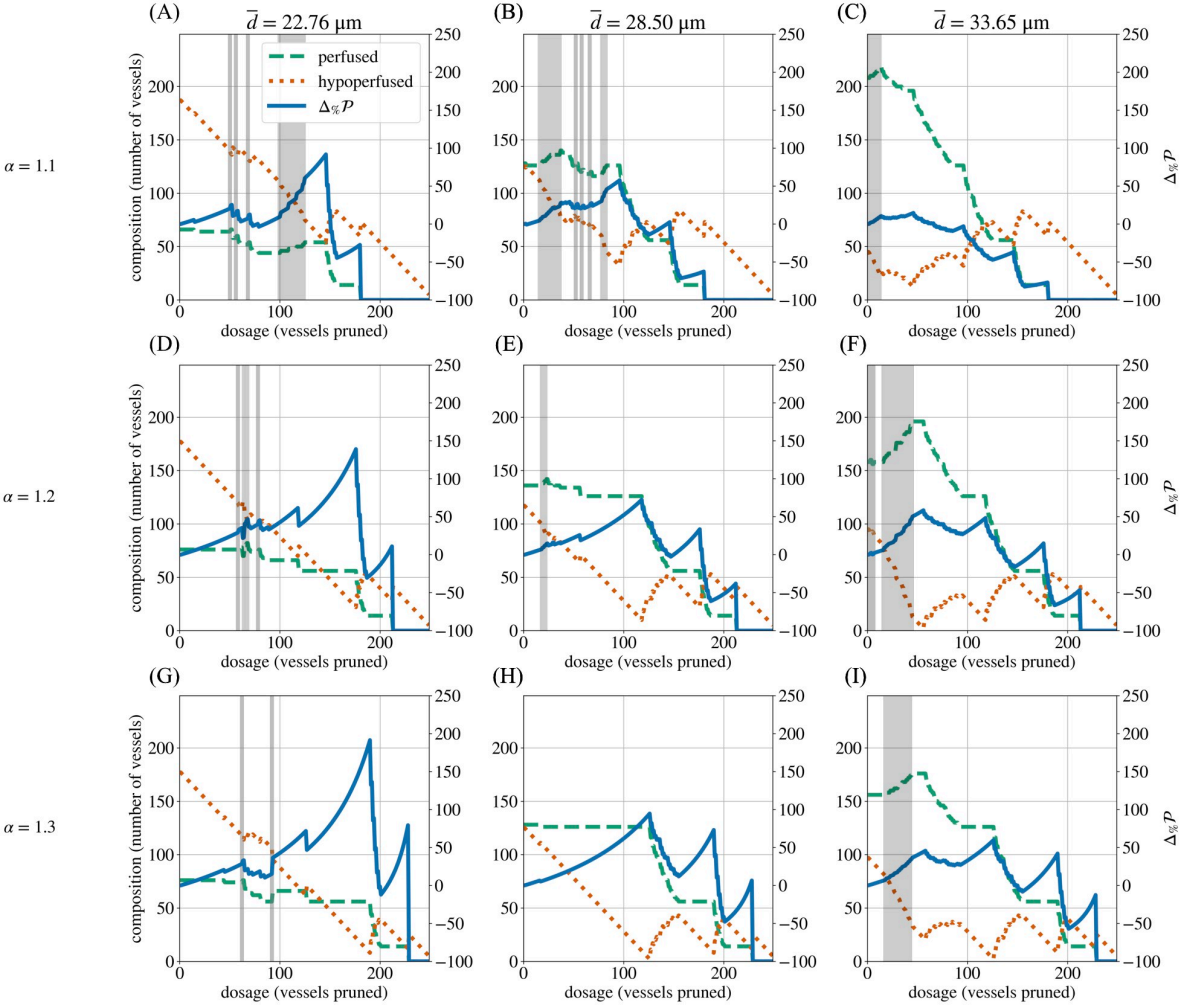
The impact of the mean diameter and the diameter heterogeneity on the perfusion response. Series of plots (A–I) showing, for the forking network, the influence of the mean diameter ($\bar{d}$) and the diameter heterogeneity (*α*) of the unpruned network on how perfusion ($\Delta_{\%}P$) changes during pruning. An increase in the number of perfused vessels is evidence of flow being rerouted into vessels that were previously hypoperfused. An increase in $\Delta_{\%}P$ without an increase in perfused vessels is evidence of Mechanism 2. Regions in which Mechanism 1 is primarily active are highlighted in grey.

Having identified two mechanisms that can increase the perfusion fraction, we next examined how the mean vascular diameter in the unpruned networks ($\bar{d}$) affects the percentage change in the perfusion fraction ($\Delta_{\%}P$). As in the biological experiments, we found that lower values of $\bar{d}$ generated larger increases in $\Delta_{\%}P$ and higher values of $\Delta_{\%}^{\text{max}}P$ (Fig 10). However, we also examined the two mechanisms of improvement in isolation and found that higher values of $\bar{d}$ were conducive to flow rerouting, while lower values tended to improve $\Delta_{\%}P$ through Mechanism 2.

**Fig 11:**
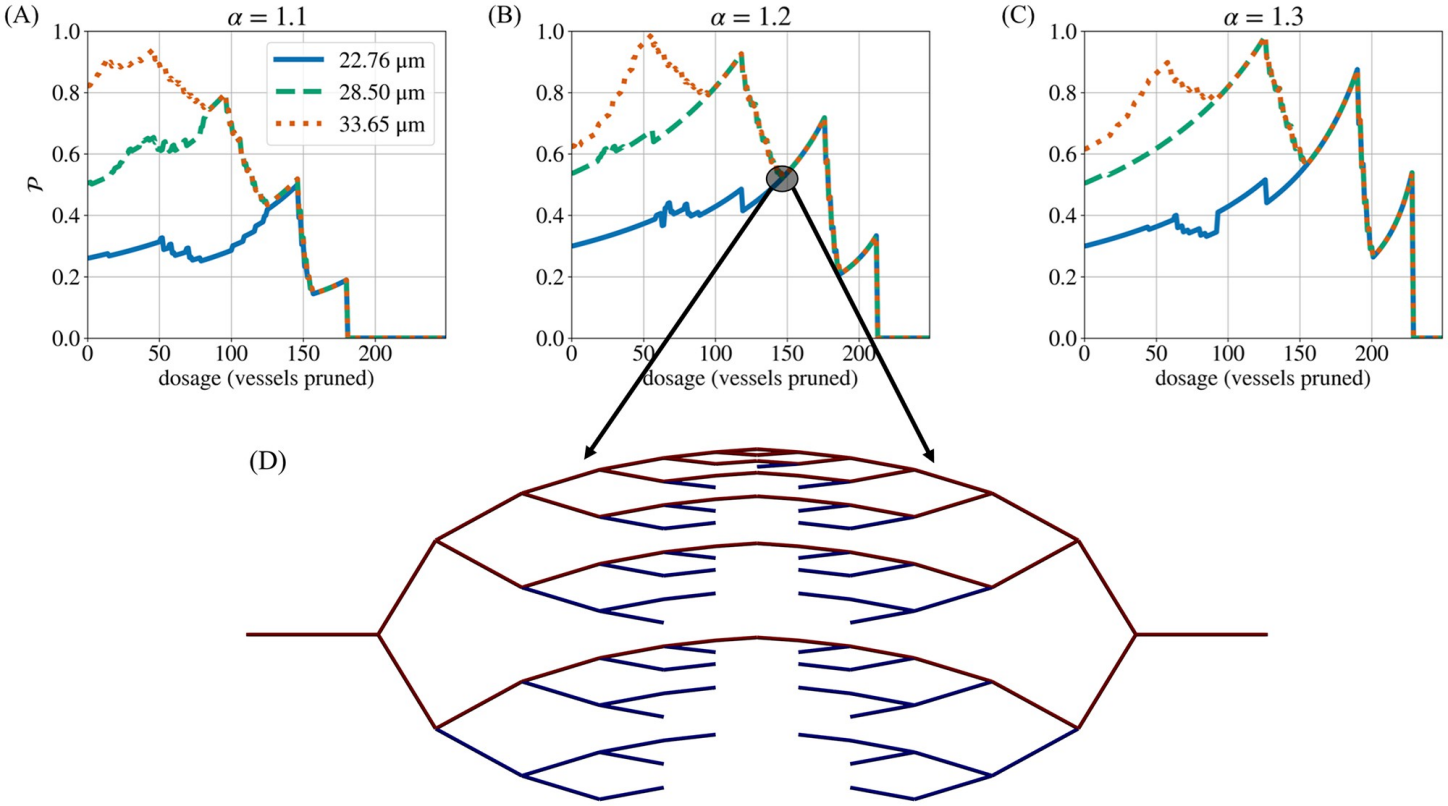
Perfusion response as measured by P. The initial perfusion fraction ($P_{0}$) depends on the vascular architecture (A–C). Note that, for a given diameter heterogeneity (*α*), the perfusion fraction (P) of all mean diameters ($\bar{d}$) converges during the later stages of pruning, when the networks only contain thicker (low-resistance) vessels. Panel (D) shows the pruned architecture at a stage common to all values of $\bar{d}$ for *α* = 1.2 (147 pruned vessels). Here, we observe that all remaining vessels are either perfused or have blunt ends (no flow). No flow rerouting can occur in the hypoperfused vessels. Thus, subsequent pruning produces the same changes in P, regardless of the initial $\bar{d}$. Note that the vessel colour indicates the local flow rate, with the range of the colourbar adjusted so that perfused vessels are dark red and vessels with little or no flow are dark blue.

**Fig 12:**
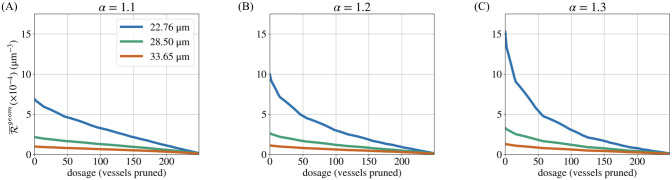
Network resistance. Since vessel conductivity depends on the fourth power of the mean network diameter (see Eq (3)), thinner forking networks offer much greater resistance (${\bar{R}}^{geom}$) to blood flow regardless of *α* (A–C). ${\bar{R}}^{geom}$ decreases monotonically during pruning because vessels are removed in order of increasing diameter.

A low $\bar{d}$ results in an increase in $\Delta_{\%}P$ for two reasons. Firstly, thinner networks have lower starting perfusion fractions ($P_{0}$) than thicker networks (Fig 11). Therefore, any change in P is greater relative to the network’s initial $P_{0}$. $P_{0}$ is lower in thinner networks because they offer greater resistance to blood flow (Fig 12). Secondly, thinner networks have a greater proportion of hypoperfused vessels that can be pruned before any perfused vessels are pruned. In these networks, P can increase a greater deal as a result of pruning only hypoperfused vessels.

On the other hand, vascular architectures with a high $\bar{d}$ are more susceptible to increases in $\Delta_{\%}P$ through blood flow rerouting (Fig 10). For these architectures, flow rates in the pruned (hypoperfused) vessels are closer to the perfusion threshold. Therefore, these vessels require a smaller increase in blood supply (via rerouting) to turn them into perfused vessels. These vessels also provide less resistance to the blood that is rerouted into them once pruning begins.

### A drop in the proportion of loops precedes a rise in perfusion

**Fig 13:**
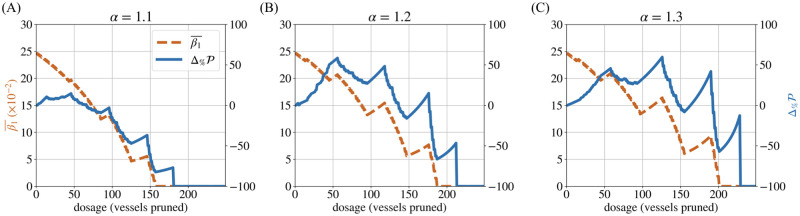
The proportion of loops. A drop in the number of loops per vessel ($\bar{\beta_{1}}$) precedes a positive change in the perfusion fraction ($\Delta_{\%}P$) in the forking networks ($\bar{d}=33.65$ μm) across all values of *α* (A–C). Minor differences in $\bar{\beta_{1}}$ arise between heterogeneities because *α* changes the order of pruning by virtue of changing the diameters of vessels.

Thus far, we have compared networks that differ in terms of their initial, unpruned architecture. Since pruning produces blunt ends (thereby changing the number of loops as discussed in the section **Topological determinants of perfusion**), the pruned networks can serve as proxies for networks that initially contain different proportions of angiogenic sprouts. Contrasting the number of loops per vessel ($\bar{\beta_{1}}$) with the change in perfusion fraction ($\Delta_{\%}P$), we observed two distinct results (Fig 13).

In the early stage of pruning (less radiation-induced cell death), $\Delta_{\%}P$ typically increases regardless of the behaviour of $\bar{\beta_{1}}$. In this stage, pruning removes the thinnest vessels, which tend to constitute hypoperfused loops or blunt ends. Thus, $\Delta_{\%}P$ increases through both mechanisms of improvement. In the later stages of pruning (more radiation-induced cell death), however, peaks and troughs in $\bar{\beta_{1}}$ correspond to those in $\Delta_{\%}P$. In particular, a reduction in $\bar{\beta_{1}}$ often precedes an increase in $\Delta_{\%}P$. When a vessel that separates two loops is pruned, the loops merge into one and the total number of loops decreases. At the site of the pruned vessel, a blunt end may be created. Subsequent pruning favours the removal of this blunt end due to its small diameter by virtue of previously being connected to the smallest vessel (recall Murray’s law, Eq (11)). Pruning the blunt-ended vessel does not change the total number of loops. However, it increases the proportion of loops ($\bar{\beta_{1}}$) and the resulting reduction in the hypoperfused vessel count leads to an increase in $\Delta_{\%}P$. Therefore, a decrease in $\bar{\beta_{1}}$ indicates that the network now contains a greater proportion of blunt ends (and vice versa). Thus, we find that $\Delta_{\%}P$ increases when networks with a low value of $\bar{\beta_{1}}$ are pruned. We also infer that $\bar{\beta_{1}}$ indicates when an improvement seen in $\Delta_{\%}P$ is caused by the pruning of blunt-ended vessels with no flow, rather than by rerouting.

## Discussion

In this paper, we used a computational model to investigate the influence of vascular architecture on the outcome of radiation-induced pruning. As per [15], we measured these outcomes in terms of overall network perfusion, as defined by the PF (Eq (1)). In our experimental data, we found that perfusion typically improved in vascular networks with lower mean diameters ($\bar{d}$) and fewer loops per vessel ($\bar{\beta_{1}}$). It may seem counter-intuitive for network perfusion to increase after vessels are removed in a model that excludes angiogenesis. However, our synthetic networks mirrored our observations of the experimental data. We modelled the influence of radiotherapy by assuming that pruning proceeds from the smallest to largest diameter vessels. Using our *in silico* model, we elucidated the mechanisms underlying the perfusion response, including two mechanisms of improvement in the PF. We have also shown that different architectural features are susceptible to different mechanisms of improvement in the PF. In particular, we found that networks with low average diameters tend to exhibit improvements in PF via the reduction of hypoperfused vessels, while networks with large average diameters are more prone to rerouting (see the section **Mean vascular diameter determines the relative contribution of the two perfusion improvement mechanisms**). In the former, large doses of radiotherapy (i.e., more vessels pruned) seem to improve the PF, while rerouting seems to occur in the latter with smaller doses of radiotherapy (Fig 10).

Therefore, our model represents a first step towards understanding the different perfusion responses found in the literature, where some studies reported increased perfusion post-irradiation and some did not [19]. Moreover, our exposition of the different mechanisms of PF improvement may explain why Bussink et al [22] observed an increase followed by a decrease in tumour perfusion. Bussink et al. attributed the increase in perfusion to the redistribution of blood and the refilling of previously non-functional vessels (in line with our model’s predictions).

While we are not aware of any experimentally informed computational models of radiotherapy-induced pruning, several studies have modelled the effect of vessel pruning due to low wall shear stress. For example, when modelling angiogenesis, Owen et al. found that poorly-perfused vessels tended to be pruned and that new vessels were formed by ‘stealing’ flow from other vessels [26]. Although we pruned our synthetic networks in order of increasing vessel diameter, we note that the thinnest vessels are likely to have the lowest blood flow. Further, our models also exhibited flow rerouting, with previously hypoperfused vessels becoming perfused following vessel pruning. Stéphanou et al. simulated vessel pruning based on flow rates to investigate the rate of drug uptake [65]. Pruning vessels randomly sometimes resulted in improved rerouting of blood flow, and increased drug uptake [65]. Pruning low-flow vessels, on the other hand, did not improve drug uptake but improved delivery speed as blunt-ended vessels were removed [65]. In our study, the removal of blunt ends is also unlikely to result in improved oxygenation, although a full investigation is reserved for future work.

### Experimental implications

Our findings also have several implications for experimental methodologies used to study radiation-induced pruning. For one, our results in the section **Two mechanisms can increase the perfusion fraction post-irradiation** have shown that the PF is not an ideal measure of a tumour’s perfusion, since it fails to account for hypoperfused vessels that are pruned. Furthermore, the PF offers no indication of a vascular network’s ability to sufficiently deliver oxygen in tissue. Theoretically, a region could feature a single perfused vessel and have a PF of 1 despite the fact that the vessel may not cover a sufficient area or carry enough red blood cells to maintain a normoxic environment. Experimentally, Bussink et al. found discrepancies between perfusion and hypoxia [22]. Monitoring the PF alone cannot tell us which mechanism (as discussed in the section **Two mechanisms can increase the perfusion fraction post-irradiation**) is at work during pruning. Discerning the mechanism is important because it tells us whether or not blood flow has improved in the remaining vessels. Future experiments could be used to validate the model’s findings. For example, if a vascular network has a low average diameter and a low number of loops per vessel, our study suggests that the network is likely to show an improvement in perfusion after irradiation. To avoid the pitfalls of using the perfusion fraction metric, it would be useful to measure flow as total blood flow rate or in terms of the tissue area under normoxic oxygen tension.

In the section **A drop in the proportion of loops precedes a rise in perfusion**, we showed that the number of loops per vessel may act as an indicator of improvements in PF via the reduction of hypoperfused vessels. In Table 1, we classified biological tumours into groups A and B based on the ΔP on a fixed day. However, the non-monotonic response of P in our simulations implies that this classification is subject to the sampling point (Table 4). In other words, a network belongs to group A or B depending on the number of vessels pruned from it. Interestingly, for any fixed *α*, the lower the mean diameter, the more often one observes an increase in PF. Similar correlations were found in real vasculatures.

**Table 4:** The classification of forking networks into groups A and B varies based on the sampling point, i.e., how many vessels have been pruned when $\Delta_{\%}P$ is measured. Cells have been shaded green or red to represent positive or negative values of $\Delta_{\%}P$.

Dosage (vessels pruned)	$\bar{d}=22.76$ μm			$\bar{d}=28.50$ μm			$\bar{d}=33.65$ μm		
	*α* = 1.1	*α* = 1.2	*α* = 1.3	*α* = 1.1	*α* = 1.2	*α* = 1.3	*α* = 1.1	*α* = 1.2	*α* = 1.3
25	7.13	10.48	10.48	15.66	12.11	8.75	8.36	16.07	13.31
50	20.26	24.02	20.76	22.08	22.20	22.08	8.52	53.85	39.92
75	-1.49	37.62	15.30	28.09	30.95	39.13	-6.23	39.55	32.28
100	9.52	42.67	42.67	38.62	52.21	61.72	-14.70	31.01	32.69
125	60.46	44.51	70.32	-14.20	41.32	93.06	-47.20	21.65	58.41
150	17.95	79.25	79.25	-35.38	0.17	21.63	-60.23	-13.78	-0.20
175	-32.07	135.98	135.98	-64.97	31.87	40.11	-78.44	13.51	14.96
200	-100.00	-13.69	-1.36	-100.00	-51.77	-41.44	-100.00	-58.49	-51.95
225	-100.00	-100.00	60.71	-100.00	-100.00	-4.58	-100.00	-100.00	-21.71

**Fig 14:**
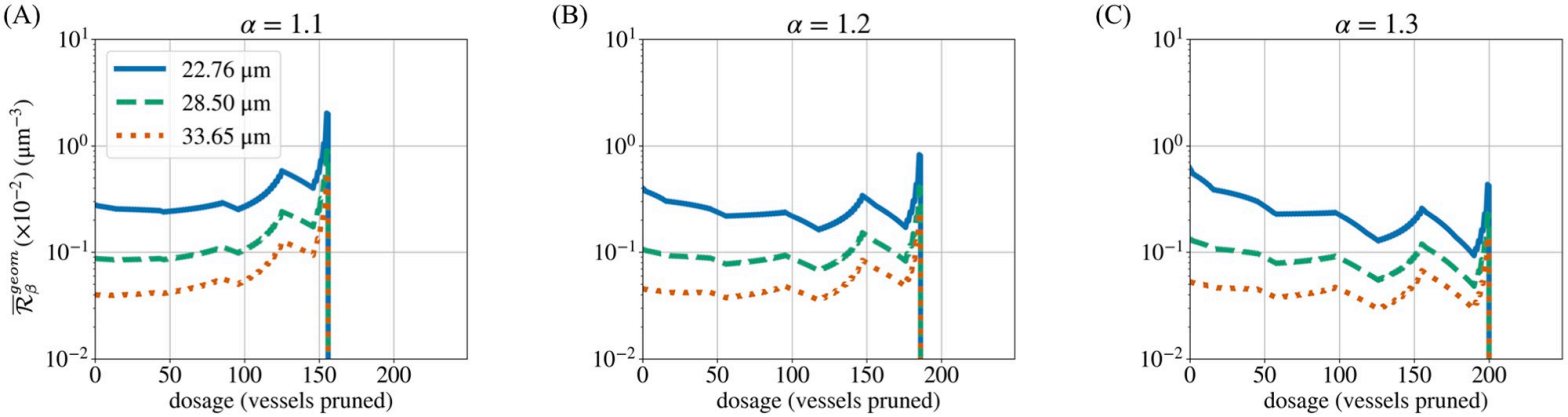
Resistance per loop. The resistance per loop (${\bar{R}}_{\beta }^{geom}$) follows trends inverse to those observed for the loops per vessel ($\bar{\beta_{1}}$) for all values of *α* (A–C), but can also distinguish between networks of varying mean diameter.

We also computed for forking networks a compound metric discussed in the section **Metrics combining geometry and topology**: the resistance per loop. As expected from Eq (10), over the course of pruning, the trend of ${\bar{R}}_{\beta }^{geom}$ follows that of $\bar{\beta_{1}}$ in an inverse fashion (Figs 13 and 14). However, ${\bar{R}}_{\beta }^{geom}$ also encodes information on the mean diameter (via resistance) and thus allows us to distinguish between networks of varying mean diameters. Consistent with real vasculatures, we observe that networks with initially higher ${\bar{R}}_{\beta }^{geom}$ exhibit greater potential for perfusion improvement, as measured by $\Delta_{\%}^{\text{max}}P$ (compare with Fig 10). This leads us to propose the metric ${\bar{R}}_{\beta }^{geom}$ for future investigations as a predictor of perfusion improvement, useful especially when $\bar{d}$ and $\bar{\beta_{1}}$ individually give contradictory predictions. This might be relevant, for example, for vasculatures with low mean diameters and a large proportion of loops, even though such a combination of characteristics did not occur within the small tumour sample studied here. These speculations need to be confirmed by future work and the mechanism of improvement must also be discerned.

While the focus of this study is the generation of mechanistic insights into enhanced perfusion, we note that the clinical implications of our model are limited by current vascular imaging technologies. High-resolution methods, such as the multiphoton microscopy used in this study, are limited to a penetration depth of approximately 1 mm, requiring the use of a window chamber [66]. In a clinical setting, vascularised tumours may be located below this depth. On the other hand, techniques such as MRI and CT have previously been used to study vascular function in humans but are limited to a resolution of 100 microns, while our model shows that the redirection of blood flow is prevalent in smaller vessels [67]. Thus, our predictions from synthetic networks would require clinical imaging technology to advance considerably before their prognostic value in humans can be evaluated.

### Future work

In this work, we used forking and hexagonal networks to illustrate the role of hierarchy in vascular architecture. However, both network types are regular (i.e., symmetric) and might not adequately represent all features of dysregulated tumour vasculature. As a next step, one should consider irregular networks, such as those generated by the Voronoi tessellation of a planar region [68, 69]. Tumour vasculature is further known to contain many leaky and tortuous vessels which will also need to be accounted for in the model.

We performed our blood flow simulations under the assumption that the haematocrit (fraction of blood made up of red blood cells) distributes itself evenly in all vessels. In reality, however, the spread of haematocrit at vessel junctions is more complex. The haematocrit in each vessel affects its flow rate (Eq (17)), as well as its capacity to carry oxygen. Additionally, our colleagues have hypothesised that irradiating a tumour with a larger proportion of small vessels might be more likely to lead to improved perfusion by altering the proportion of haematocrit splitting [15]. Therefore, it will be important to contrast different haematocrit splitting rules sourced from the literature [23, 70, 71]. Different splitting rules will give rise to different oxygen distributions in the tissue. These distributions are a key determinant of the efficacy of standard oncological treatments, such as chemotherapy, which can also be modelled within Microvessel Chaste [62, 63] as a chemical transport process. The death of tumour cells caused by radiotherapy or chemotherapy lowers net oxygen consumption, which further increases tissue oxygen levels. The inclusion of an oxygen consumption term proportional to the local density of live tissue cells will be an important next step in the precise modelling of tissue oxygen perfusion. We have already highlighted that the PF is an imprecise metric to describe the oxygenation status of the tissue. Once we explicitly simulate oxygen diffusion, we will need to adopt and develop new spatial metrics that better describe the spread of oxygen. With a model of oxygenation in place, we will then consider the dynamic nature of the tissue perfused by the vasculature. Cells that comprise the tissue will have varying uptake rates, and will divide and die based on the amount of oxygen available to them.

Pruning can also be conducted stochastically, provided a reliable relationship between the vessel diameter and the probability of its pruning is found, and the pruning rules can be modified to reflect radioresistance when the region is anoxic. Let us note that the existing model can also be adapted to better represent the regression of hypoperfused vessels as observed in developmental vascular networks by pruning vessels in order of increasing flow rate [17, 49]. Since the vasculature itself is also dynamic, future models must factor in structural adaptation and the secretion of angiogenic factors. Random sprouting models of tumour angiogenesis may be useful here [72]. Percolation models would also serve as a good representation of the statistical distribution of avascular spaces found in tumours [73]. Moreover, percolation models have been used to replicate observed oxygenation conditions in mice tumour xenografts [73]. Such a study may also involve modifying haematocrit splitting rules to include higher-order splitting, since tumour vasculature is not limited to bifurcations [23].

Finally, we may leverage our insights from synthetic networks and apply them to a real biological network obtained via the methods outlined in [35]. Provided that inlet and outlet vessels with appropriate boundary conditions are identifiable, we may then simulate blood flow and the pruning of vessels in image-derived networks. However, the implications of this study remain limited due to a relatively small experimental dataset associated with a single tumour cell line. Trends identified in this work will thus need to be tested using different cancers and biological models.

## Materials and methods

### Experimental procedures and data preprocessing

#### Ethics statement

All animal experiments were conducted in accordance with the United Kingdom Animals (Scientific Procedures) Act 1986 as amended (Amendment Regulations 2012 [SI 2012/3039]), under the authority of a UK Home Office Project License (PPL 30/2922 and PCDCAFDE0), with local ethical approval from the University of Oxford Animal Welfare and Ethical Review Panel, as previously described [15].

#### Experimental procedures

**Abdominal imaging window implantation**. This procedure was based on a previously described method [74]. Transgenic mice with fluorescent protein tdTomato expressed in endothelial cells on C57Bl/6 background were prepared in a surgical unit, and administered with inhalational anaesthesia and pre-operative analgesics. Body temperature and respiration rates were monitored throughout the procedure. A 1 cm cut was made along the abdominal midline approximately 5 mm underneath the sternum followed by blunt dissection around the cut to separate the connective tissue from the skin. A custom-made imaging window frame (Workshop at the Department of Oncology, Oxford University) was fitted underneath the skin. Continuous sutures were used to secure the skin to the window frame. Approximately 2.5 × 10^5^ MC38 cells (murine colon adenocarcinoma cells) stably expressing eGFP in 5 μL containing 30% of Matrigel and 10% of Evan’s blue dye were injected under the connective tissue and above the abdominal muscle layer. The chamber was then flushed with water to lyse non‑injected cells by osmotic shock, tapped dry with sterile cotton swabs and flooded with saline. A cover glass glued on the chamber’s lid was secured onto the window frame. The animals were then placed onto a heat mat for post‑operative recovery, and their health and tumour growth was monitored by visual examination.

**Treatment regimes**. Animals with tumours approximately 100 mm^3^ growing in the window chamber were administered radiation treatment. Prior to the radiation treatment, mice were anaesthetised under inhalation with isoflurane and placed in an imaging-guided small animal radiation research platform (SARRP) irradiator (Xstrahl Ltd). A Cone Beam CT scan (computerised tomography) of each mouse was obtained and the treatment was planned using Muriplan (Xstrahl Ltd). The SARRP was used to deliver 15 Gy of X-rays (220 kVp copper filtered beam with HVL of 0.93 mmCu) to the tumour at 2 Gy per minute. Dosimetry of the irradiator was performed as previously described [75]. We refer to the start of treatment as Day 0.

**Intravital two-photon imaging**. Mice were imaged for 7 days after radiation treatment with a Zeiss LSM 880 microscope equipped with a respiratory monitoring system. The stage and atmosphere were heated to 37°C. To label perfused vessels, Qtracker705 Vascular Labels (0.2 μM, ThermoFisher Scientific) were infused intravenously using a motorised pump at a rate of 0.84 μL⋅min^−1^. A mode-locked MaiTai laser tuned to 920 nm was used to simultaneously excite eGFP, tdTomato, and Qtracker705. The Qtracker705 signal was acquired through a BP700/100 filter with a non-descanned detector. GaAsP detectors were used to acquire the signal of tdTomato selected by a BP 650/45 filter and the eGFP selected by a BP525/50 filter. Images were acquired in Z-stack tile scans with a pixel size of 0.823 μm and an image size per tile of 512 × 512 × 5 in *x*, *y*, and *z*, respectively. A water immersion 20× objective made for UV-VIS-IR transmission with a numerical aperture of 1.0 was used. Representative examples of vascular networks from our experiments are displayed in Fig 2.

#### Data preprocessing

The biological networks were obtained by multiphoton intravital 3D imaging [76] and consisted of 3D stacks of images of tumour blood vessels. Skeleton files were extracted from the imaging data by combining two segmentation models and taking their geometric average. The skeletons were then pruned (see reference [77], p. 165, for a full description). We extracted blood vessel networks from skeleton files using the method VesselTree from unet_core.vessel_analysis in the Python code package unet-core [78]. The extracted networks consist of points on vessel branches (multiple points per vessel branch including branching points) which represent the network nodes, and the vessels that connect them which constitute the edges of the network. VesselTree also enables us to extract network features such as vessel diameters and lengths.

### Simulation methods

#### Blood flow

We assume that the blood flow rate *Q* in a vessel of length *L* and diameter *d* is determined by Poiseuille’s law (Eq (3)). Following [23, 53], the effective blood viscosity in Eq (3) is expressed as:

$$\begin{array}{c} \mu^{\text{eff}}=\mu_{p}[1+\left(\mu_{45}-1\right)(\frac{{\left(1-H\right)}^{C}-1}{{\left(1-0.45\right)}^{C}-1})(\frac{d}{d-1.1}{)}^{2}][\frac{d}{d-1.1}{]}^{2}, \end{array}$$ (17)

where *H* is the discharge haematocrit, *d* is the vessel diameter, *μ*_*p*_ is the plasma viscosity,

$$\begin{array}{c} \mu_{45}=6e^{-0.085d}+3.2-2.44e^{-0.06d^{0.645}}, \end{array}$$ (18)

and:

$$\begin{array}{c} C=\left(0.8+e^{-0.075d}\right)(-1+\frac{1}{1+10^{-11}d^{12}})+\frac{1}{1+10^{-11}d^{12}}. \end{array}$$ (19)

As discussed in the section **Geometric determinants of perfusion**, we assume that haematocrit is distributed evenly within the network (*H* = *H*_*inlet*_ = 0.45) to yield a linear flow problem. With signed flow rates *Q*_*i*_ for each vessel *i* (i.e., each edge connected to a node), we also impose conservation of blood at each network bifurcation node:

$$\begin{array}{c} \sum_{i}Q_{i}=0. \end{array}$$ (20)

In each (unpruned) network, the leftmost nodes serve as inlets and all other vessels with one detached node serve as outlets. Inlet nodes are assigned a pressure (*p*_*inlet*_) of 3333 Pa (≈ 25 mmHg), while outlet nodes are assigned a pressure (*p*_*outlet*_) of 2000 Pa (≈ 15 mmHg) [79].

#### Software development and post-processing

We performed our simulations using Microvessel Chaste (version 3.4.3), an add-on to the open-source simulation package Chaste (version 2020.1) [62, 63]. We added custom functionality to the base version of Microvessel Chaste, including new network generators and pruning functions. We generated synthetic networks detailed above and pruned them vessel-by-vessel in order of increasing vessel diameter. At each pruning step, we updated the flow distribution, and exported the perfusion fraction and the network itself in the .vtk format, accounting for all vessels that had not yet been pruned. We used Python scripts to generate from the .vtk files adjacency matrices weighted by vessel diameter and vessel length, from which we calculated key geometric metrics, such as mean diameter and mean geometric resistance. Based on a MATLAB package [80], we wrote Python scripts to import the adjacency matrices from above, calculate both the number of loops and loops per vessel at each pruning stage using Eq (6), and finally divide the mean resistance by the latter.

## Supporting information

S1 Appendix Sections S1.1-S1.8 for *“Enhanced perfusion following exposure to radiotherapy: a theoretical investigation”*. (PDF)
