# Supplementary material for: Enhanced perfusion following exposure to radiotherapy: A theoretical investigation
Source: PLoS Comput Biol. 2024 Feb 16;20(2):e1011252. doi: 10.1371/journal.pcbi.1011252 (PMC10903964; doi:10.1371/journal.pcbi.1011252)
Supplement: S1 Appendix — (PDF) [file pcbi.1011252.s001.pdf]

Enhanced perfusion following exposure to radiotherapy: A theoretical investigation

S1 Appendix

Jakub Köry[1,2,a,*] Vedang Narain[2,a] Bernadette J. Stolz[2,3] Jakob Kaeppler[4]
Bostjan Markelc[4,5] Ruth J. Muschel[4] Philip K. Maini[2] Joe M. Pitt-Francis[6]
Helen M. Byrne[2]

[1]School of Mathematics and Statistics, University of Glasgow, Glasgow, United Kingdom
[2]Mathematical Institute, University of Oxford, Oxford, United Kingdom
[3]Laboratory for Topology and Neuroscience, École Polytechnique Fédérale de Lausanne, Lausanne, Switzerland
[4]Cancer Research UK and MRC Oxford Institute for Radiation Oncology, Department of Oncology, University of Oxford, Oxford, UK
[5]Department of Experimental Oncology, Institute of Oncology Ljubljana, Ljubljana, Slovenia
[6]Department of Computer Science, University of Oxford, Oxford, United Kingdom
[a]Equally contributing authors
[*]Corresponding author: jakub.koery@glasgow.ac.uk

S1.1 Reliability of the data as measured by the number and size of connected components

As noted in the section **Understanding perfusion response to radiotherapy and its determinants** of the main body, the number of vessels for tumour vasculature 7 increased throughout the first four days post-irradiation. Table A documents that this vasculature had, relative to the network size, an extremely large number of connected components (CC) and an extremely small largest connected component, indicating problems in image processing, which is why this tumour was excluded from subsequent analysis.

Table A: Statistics relating to the number and size of connected components indicate problems with image acquisition for tumour number 7.

**Tumour statistics on Day 0**	**1**	**2**	**3**	**4**	**5**	**6**	**7**
Vessel count (network size)	1000	4087	1038	2175	644	528	436
Number of CCs β_0	301	184	173	301	82	86	184
Number of CCs per size $\overline{\beta_0}$	0.30	0.05	0.17	0.14	0.13	0.16	0.42
Size of largest CC	168	3467	317	1365	338	353	59
Size of largest CC per size	0.17	0.85	0.31	0.63	0.52	0.67	0.14

S1.2 Fitting the forking network and assigning vessel IDs

S1.2.1 Adjusting vessel diameters to match summary statistics for real vasculatures

We fix the number of generations at 7 throughout this work so that the vessel diameters span a sufficiently large range. In order to generate significant intra-generational diameter heterogeneity we consider three values of α, namely 1.1, 1.2, and 1.3. Considering the smallest and the largest α, the means and standard deviations of the diameter distributions lie within the ranges delineated by summary statistics for the real vasculatures if and only if the inlet diameter is roughly in the range $80-100$ μm (see Fig A). Taking the inlet

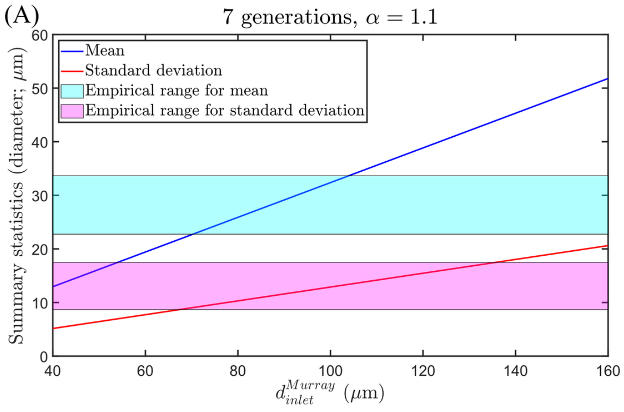

Fig A: The means (blue) and standard deviations (red) of diameter distributions in Murray's law forking networks with 7 generations of vessels as a function of the inlet diameter (d_{inlet}^{Murray}) for (A) $\alpha = 1.1$ and (B) $\alpha = 1.3$. The coloured areas delineate ranges of means (cyan) and standard deviations (magenta) of diameter distributions found in real vasculature.

diameter to be 90 μm, the top panels in Fig B show bar charts documenting the diameter distributions for varying α, while the bottom panels detail how the diameters are distributed within individual generations. Note from Figs A and B that both increasing the inlet diameter for a fixed α and increasing α for a fixed inlet

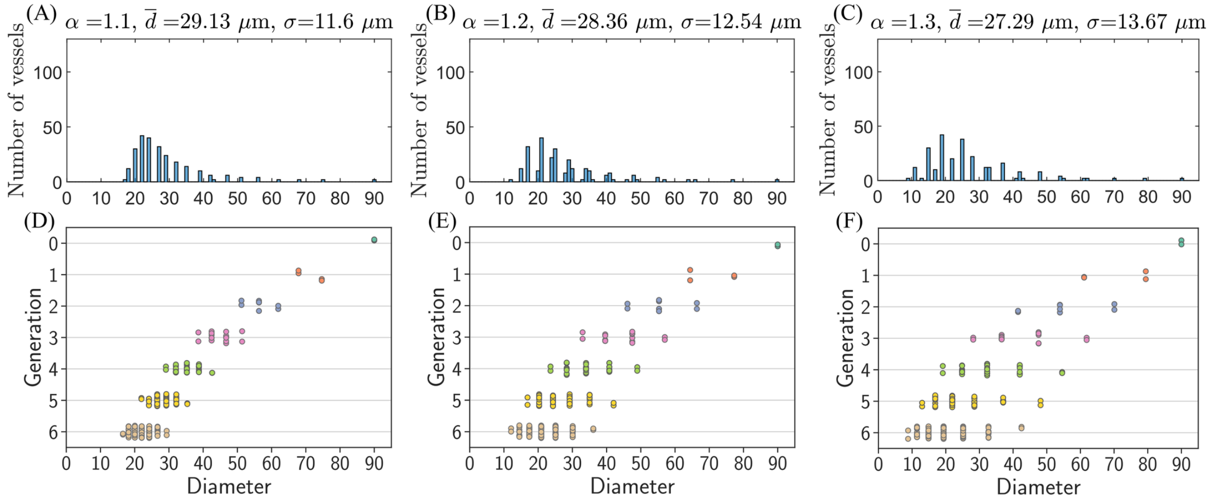

Fig B: The parameter α dictates the diameter distribution of the Murray's law forking networks. The resulting distributions are depicted in the form of (A–C) histograms as well as (D–F) swarm plots in which vessels belonging to the same generation are of the same colour.

diameter lead to an increase in standard deviation. However, both approaches also yield an altered mean

diameter. As we wish to study the impact of the mean and the standard deviation on network perfusion (as affected by radiotherapy-induced vessel pruning) independently, we proceed in a different manner.

We aim to find a new value for the inlet diameter to obtain realistic standard deviations for all studied values of α. We also aim to offset the diameters of all vessels in the Murray's law forking networks (i.e., networks obeying Eq (11)) by the same constant to match the mean diameter in real vasculatures, namely the smallest (22.75 µm), average (28.5 µm), and largest (33.65 µm) mean diameters. Following Fig B, this simply means that all data points in strip plots and all columns in histograms will be shifted right or left to match the means observed in real data. Note that this offsetting does not alter the standard deviation. On the other hand, for a fixed offset, α only modulates the standard deviation.

After specifying the number of generations, α and the offset, all vessel diameters follow from the inlet diameter. To limit the possible range for the inlet diameter, we require that for all three values of α, the Murray's law forking networks manifest standard deviations within the experimentally-observed range (from 8.68 µm for the least to 17.49 µm for the most heterogeneous vasculature). From Fig A, we thus conclude an approximately $70 - 110$ µm range for the inlet diameter of the Murray's law forking network. Finally, while we have designed our offset forking networks so that they match the means and standard deviations found in real vasculatures, we must make sure that we do not assign any unrealistically small — or even negative — diameters (for all values of α). In Fig C, we plot the minimum vessel diameter as a function of the inlet diameters for two extreme values of α (1.1 and 1.3) and for the three offsets used in our simulations. The value $d_{inlet} = 100$ µm used in [1] would result (using $\alpha = 1.3$ and offsetting to match the smallest mean diameter in real vasculatures) in vessel diameters well below both the smallest diameter found in our biological networks (≈ 7.39 µm) and even the minimum diameter of an undeformed red blood cell, i.e., 6 µm [2]. Therefore, we will use $d_{inlet} = 75$ µm as default in the Murray's law forking networks, which yields plausible vessel diameters in all scenarios.

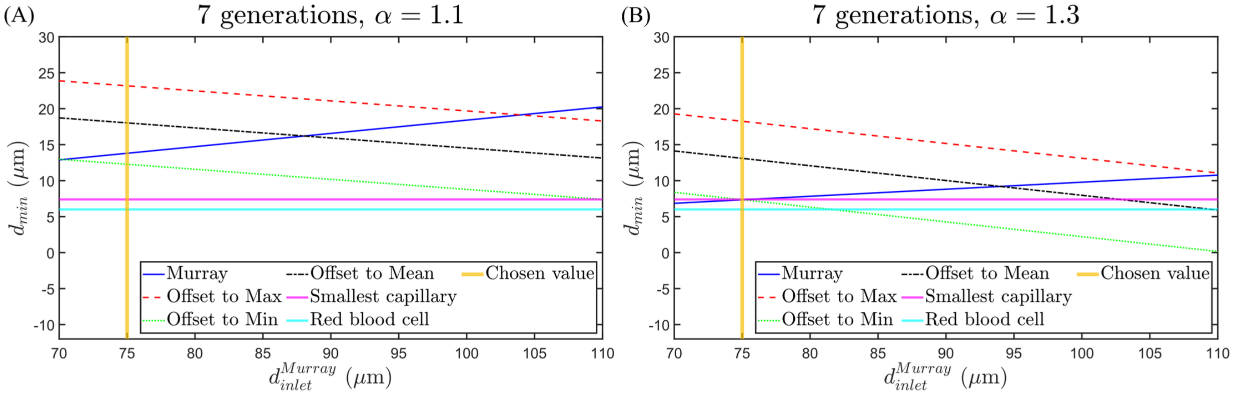

Fig C: The blue curves show minimum vessel diameter (d_{min}) as a function of inlet diameter (d_{inlet}^{Murray}) in Murray's law forking network with 7 generations of vessels for (A) $\alpha = 1.1$ and (B) $\alpha = 1.3$. The minimum diameter changes due to offsetting to match the smallest (dotted green), average (dash-dotted black), and largest (dashed red) mean diameter in real vasculatures. The diameter of the smallest vessel found in real vasculatures (≈ 7.39 µm) is indicated with horizontal magenta and the minimum diameter of red blood cells (6 µm) with horizontal cyan lines. The value of inlet diameter chosen for our simulations with 7 vessel generations (75 µm) is indicated with vertical yellow lines.

S1.2.2 Assigning vessel IDs

During vessel network generation in Microvessel Chaste, we assign each vessel a unique ID which is an integer number that will be used to determine the order of pruning in case two or more vessels have the same diameter. The way in which vessel IDs are assigned is illustrated in Fig D. In the left (diverging) half of the domain, vessels are assigned IDs that increase with increasing generation number and their mirror images in the right (converging) half of the domain are always assigned IDs equal to those of their left-half counterpart plus 2.

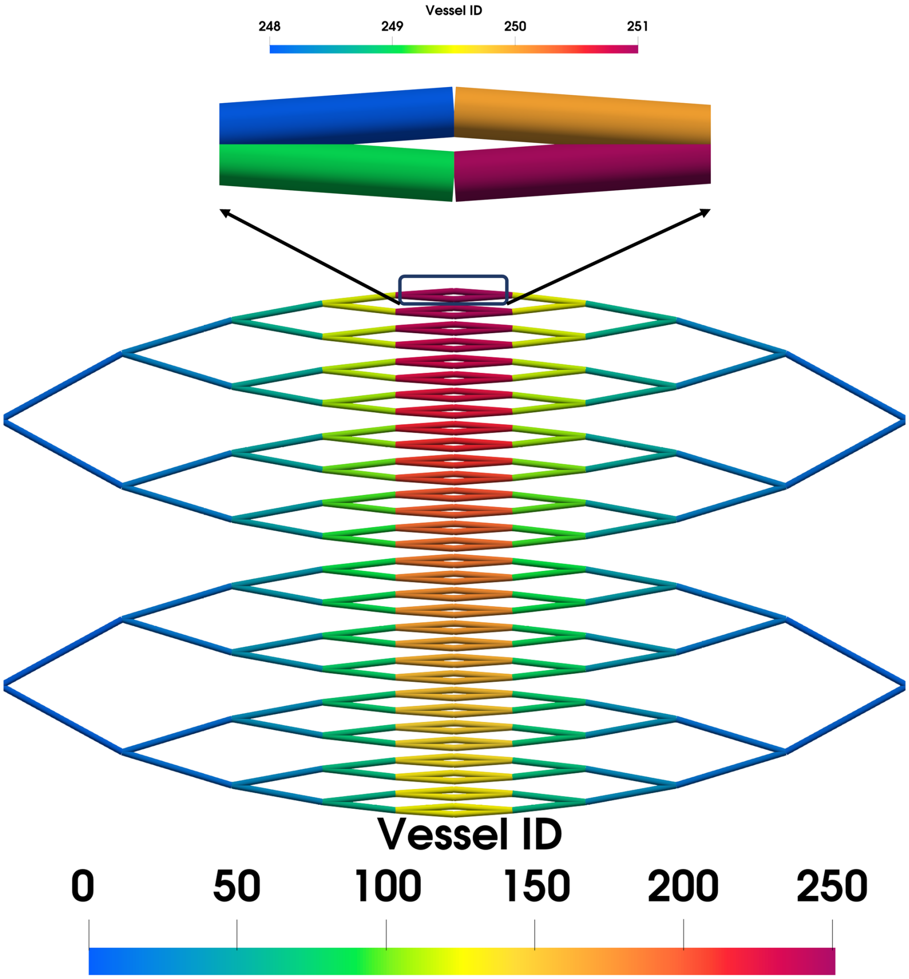

Fig D: Vessel IDs are assigned based on the order in which the vessels are added to the network during network generation, as demonstrated here. Note that the range of the colourbar in the top panel has been adjusted to demonstrate the vessel IDs assigned for two daughter vessels of the same parent vessel as well as for their mirror images in the right (converging) part of the domain.

S1.3 Design of hexagonal network

In the non-hierarchical network, regular hexagons populate a two-dimensional domain measuring 2 mm by 2 mm (Fig E). Each vessel has a length (L_{hex}) of 100 µm. The network has 11 inlets and 10 outlets. As observed in biological tumours, the distribution of vessel diameters resembles a log-normal distribution [1, 3]. Therefore, vessel diameters in our synthetic networks are randomly sampled from a log-normal distribution with the probability density function:

$$f(d) = \frac{1}{d\sigma_{\mathcal{N}}\sqrt{2\pi}} \exp\left(-\frac{(\ln d - \mu_{\mathcal{N}})^2}{2(\sigma_{\mathcal{N}})^2}\right),$$

where d is the vessel diameter,

$$\mu_{\mathcal{N}} = \ln\left(\frac{(\bar{d})^2}{\sqrt{(\bar{d})^2 + (\sigma)^2}}\right), \qquad \text{and} \qquad \sigma_{\mathcal{N}} = \sqrt{\ln\left(1 + \left(\frac{\sigma}{\bar{d}}\right)^2\right)}.$$

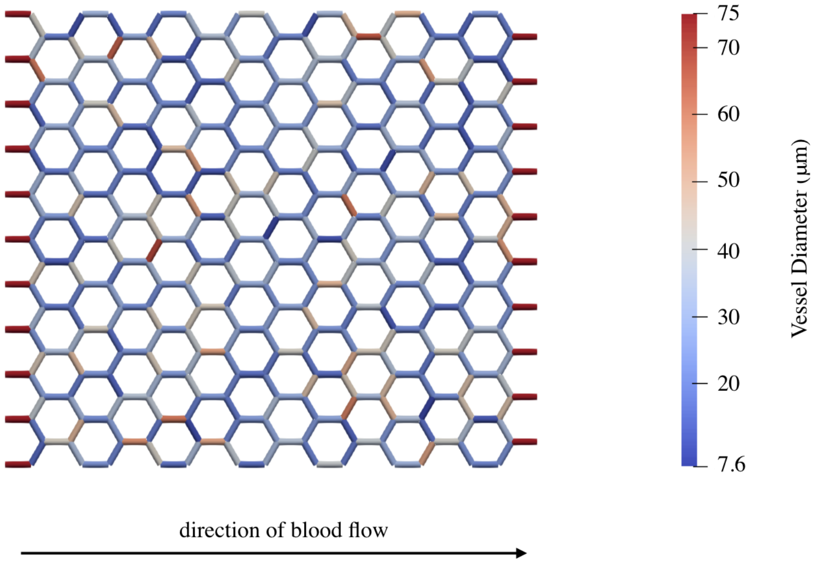

Fig E: An example of a hexagonal network with diameters taken from a log-normal distribution. Blood flows in from the leftmost nodes and out from the rightmost.

We vary the mean ($\overline{d}$) and standard deviation (σ) of the diameter distribution to cover the ranges exhibited by the biological tumours (Fig F). We also impose a minimum and maximum limit for the distribution. The minimum is set to 7.39 μm (the lowest vessel diameter observed in the experiments) and the maximum to 75 μm (the inlet diameter of the forking network). The inlet and outlet vessels are always assigned a diameter

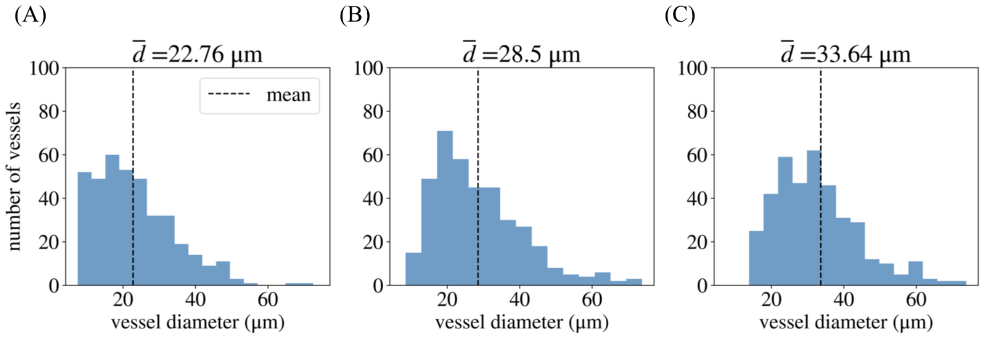

Fig F: Examples of diameter distributions (A–C) with a consistent standard deviation (σ = 13.23 μm) centred on varying mean diameters for hexagonal networks.

of 75 μm in line with the forking network to prevent them from being pruned until no other vessels remain. Results for each network configuration are averaged across 100 randomly sampled distributions.

S1.4 The effect of heterogeneity and hierarchy

Although we lack the experimental data to investigate the effects of vascular diameter heterogeneity and hierarchy in biological networks, we have investigated these features in our synthetic networks. We discuss our results below.

S1.4.1 Heterogeneity results in increased and persistent perfusion

As mentioned in the section **Network design** of the main body, diameter heterogeneity in forking networks is modulated by making one daughter vessel α-times thicker than the other. We find that as α increases, the relative improvement in perfusion fraction ($\Delta_{\%}\mathcal{P}$) also increases (Fig 10). Unlike our analysis of mean diameter ($\bar{d}$), however, the initial perfusion fractions ($\mathcal{P}_0$) of networks for any given $\bar{d}$ are similar (with the exception of $\bar{d}$ = 33.65 µm) across different values of α (Fig 12). Therefore, the initial perfusion fraction does not explain why higher levels of heterogeneity lead to a greater relative improvement. Examining the network composition helps explain this observation (Fig 10). The maximum perfusion fraction attained over pruning ($\Delta_{\%}^{\max}\mathcal{P}$) represents the point at which the ratio between the number of perfused vessels and the total number of vessels is greatest (see Eq (1)). Since the latter encapsulates the former, this point also represents the stage at which the ratio between the number of perfused and hypoperfused vessels is greatest. As α increases, the hypoperfused vessel count attains smaller minimum values and, therefore, the $\Delta_{\%}^{\max}\mathcal{P}$ reaches higher values. These successively smaller minima are reached because more heterogeneous networks feature greater proportions of small vessels, which carry blood at a flow rate below the perfusion threshold (Fig B). Therefore, there exist a greater number of hypoperfused vessels that can be pruned to raise $\Delta_{\%}\mathcal{P}$ before pruning perfused vessels.

We also note that networks with smaller values of α require fewer vessels to be pruned before zero perfusion is reached. The network exhibits zero perfusion when there is no connected path between the inlet and outlet vessels. As the diameter asymmetry induced by α increases, several vessels in the middle of the network become thicker than vessels in other generations and remain unpruned for longer. Therefore, a path between the inlet and outlet is preserved through later stages of pruning in more heterogeneous forking networks.

S1.4.2 Hierarchy is conducive to perfusion enhancement

The final architectural feature to be evaluated is network hierarchy. Our forking network exhibits diameter hierarchy in the form of a branching architecture, in which parent vessels are thicker (and longer) than their daughter vessels. This order is absent in hexagonal networks (Fig E). We observed less pronounced increases in perfusion for non-hierarchical networks than for hierarchical networks (Fig G). Inspection of the network compositions reveals that the minor increases in perfusion ($\Delta_{\%}\mathcal{P}$) are due to the removal of hypoperfused vessels rather than the rerouting of flow. As in the forking networks, $\Delta_{\%}^{\max}\mathcal{P}$ is larger in networks with lower mean diameters.

S1.5 The effect of varying haematocrit

While our simulations fix the inlet haematocrit to a value of 0.45, here we present the results of varying the haematocrit to the minimum ($H = 0.36$) and maximum ($H = 0.54$) values observed in humans [4]. From Figs H and I, we can see that lower haematocrit results in higher flow rates (as evidenced by more perfused vessels), while an increase in haematocrit results in lower flow rates (as evidenced by fewer perfused vessels). This change in flow rate is in line with our expectations from Eqs (3) and (17) describing the effect that changing the effective viscosity of the blood will have on the flow rate and how this viscosity depends on the local haematocrit. As a result, networks with a higher concentration of haematocrit show a greater increase in PF through the pruning of hypoperfused vessels.

S1.6 The effect of varying inlet diameter and number of generations

In the section S1.2.1, we fixed the number of generations in forking networks at 7 and found the inlet diameter (75 µm) which, using three offsetting scenarios, yields vessel diameter distributions with mean, standard deviation and minimum as observed in real vasculatures. Aiming to study how pruning affects perfusion in forking networks with varying number of generations, we would like to similarly find appropriate inlet vessel diameters for networks with 6 and 8 generations. Investigating first the Murray's law forking networks

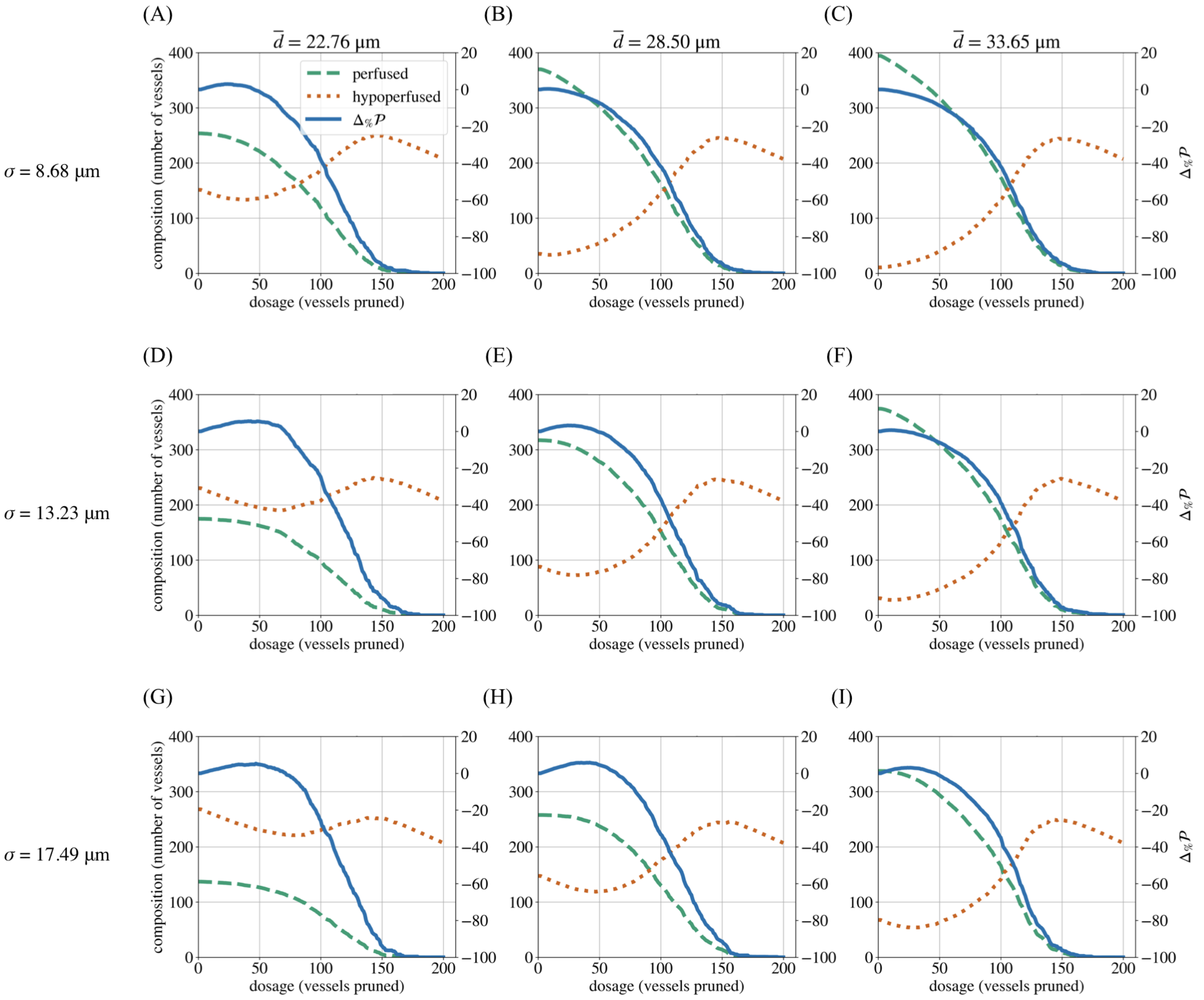

Fig G: The perfusion response ($\Delta_{\%}\mathcal{P}$) of the hexagonal network is largely monotonic during pruning, regardless of the standard deviation (σ) of the initial diameter distribution (A–I).

(recall Fig A) we find that in order for the standard deviation to match the real data, the inlet diameter must be approximately between 60 and 100 μm for networks with 6 generations and between 80 and 130 μm for networks with 8 generations (see Figs J and K).

Upon offsetting diameters to match the minimum, mean and maximum mean diameters observed in real vasculatures, we next select appropriate inlet diameters so that the smallest vessel diameter for networks offset to match the minimum mean diameter is close to the smallest diameter observed in real vasculatures (and greater than the red blood cell diameter). Based on Figs L and M, we choose the inlet diameter to be 65 μm for networks with 6, and 85 μm for networks with 8, generations.

The design of our forking network ensures that the number of vessels in successive generations doubles, with vessel diameters growing thinner in line with Murray's law (Eq (11)). Therefore, when varying the number of generations in Figs N and O, we see that a network with 8 generations has a far greater number of vessels than a network with 6 generations. Moreover, a network with thinner vessels has a greater resistance to the flow of blood and vice versa. Thus, we note that the proportion of hypoperfused vessels is greater in the network with 8 generations than in the network with 6 generations due to the same applied pressure drop across the network. The former contains more hypoperfused vessels that, when pruned, result in a greater increase in PF than the network with 6 generations. Although the network with 8 generations has a

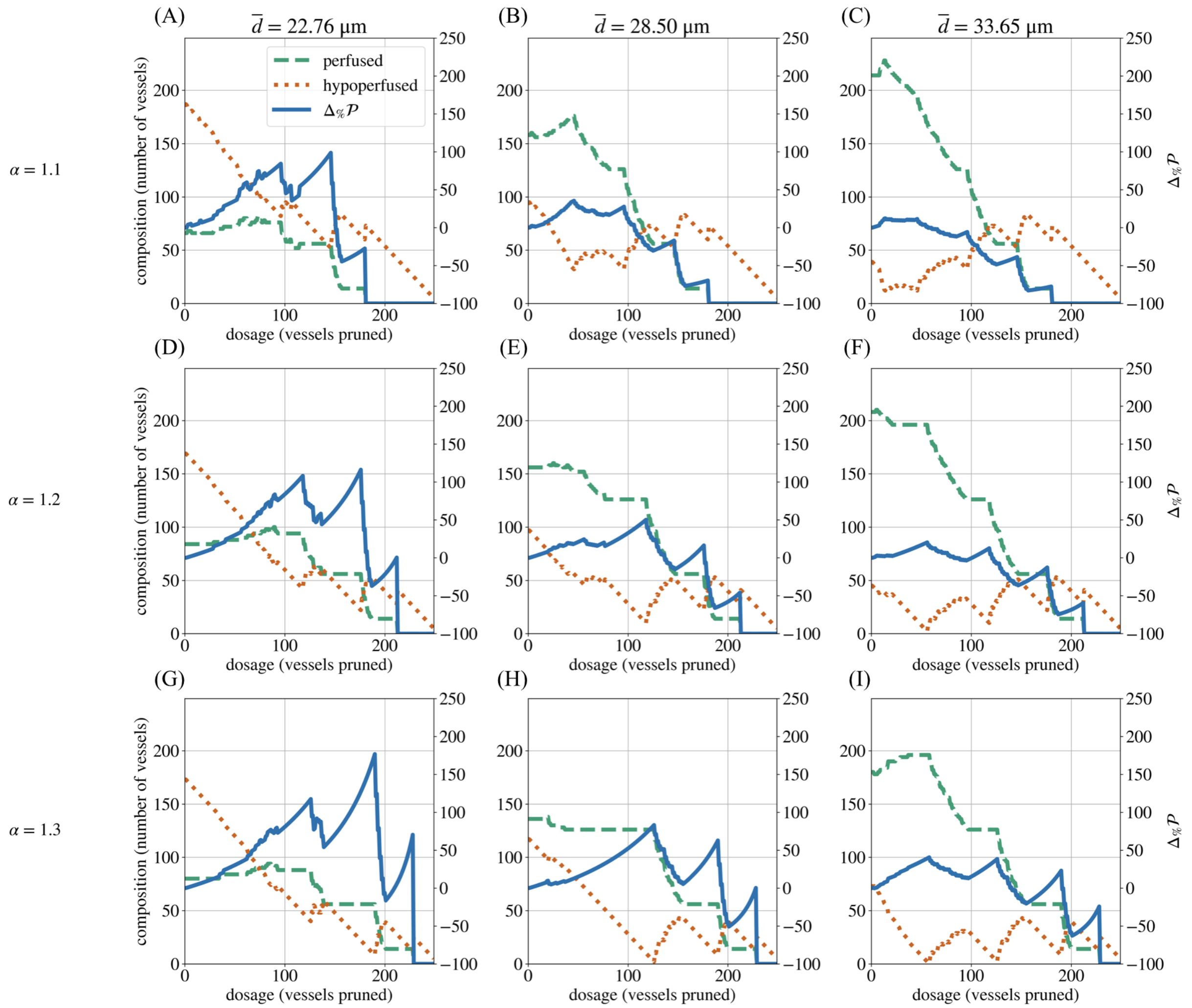

Fig H: At the lowest haematocrit concentration ($H = 0.36$), the blood flow rate is higher, resulting in a lower increase in PF through the pruning of hypoperfused vessels (A–I).

larger inlet diameter ($d_{inlet} = 85$ µm), this is insufficient to support the perfusion of the additional vessels.

S1.7 The effect of diameter threshold pruning

As outlined in the section S1.2.2, pruning vessels individually is occasionally ordered by vessel ID. To circumvent this artefact, we demonstrate here the effect of pruning vessels of similar diameters at the same time. Specifically, we set a diameter threshold and remove all vessels below that threshold. We then increase the threshold and repeat the process until the PF drops to zero. Evaluating the results, we find that the observations made from pruning vessels individually still hold, albeit with an effectively lower sampling rate, such as the greater influence of rerouting in improving the PF at larger diameters (Fig P).

S1.8 Model parameters

Parameters common to the forking and hexagonal simulations can be found in Table B. Parameters specific to the forking network can be found in Table C, while the specifications for the hexagonal network are documented in Table D.

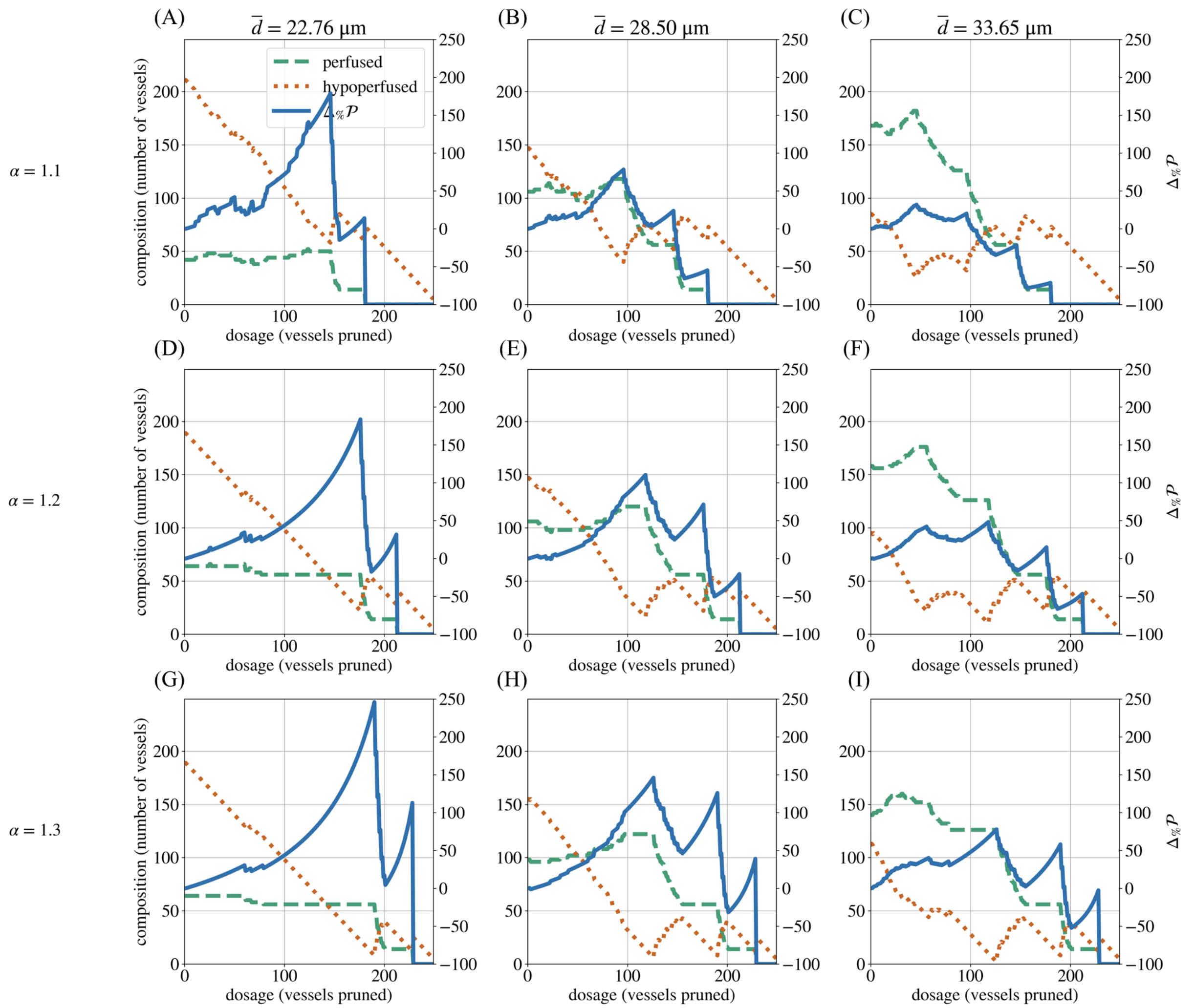

Fig I: At the highest haematocrit concentration ($H = 0.54$), the blood flow rate is lower, resulting in a greater increase in PF through the pruning of hypoperfused vessels (A–I).

Table B: Parameter values common to both forking and hexagonal simulations.

**Parameter**	**Description**	**Value**	**Unit**	**Reference**
$\overline{d}$	mean network diameter	22.76, 28.50, 33.65	µm	Section S1.2.1
d_{inlet}	inlet diameter	65, 75, 85	µm	Section S1.2.1
μ_p	plasma viscosity	10^{-3}	kg $m^{-1}s^{-1}$	[1]
H_{inlet}	inlet haematocrit	0.36, 0.45, 0.54	-	[1, 4]
p_{inlet}	inlet pressure	3333	Pa	[5]
p_{outlet}	outlet pressure	2000	Pa	[5]

Table C: Parameter values for the forking network architecture.

**Parameter**	**Description**	**Value**	**Unit**	**Reference**
λ	ratio of vessel length to diameter	4	-	[1]
α	relative thickness of daughter vessels	1.1, 1.2, 1.3	-	Section S1.2.1
Q_{min}	perfusion threshold	3 x 10^{-12}	m^3 s^{-1}	Section **Perfusion threshold**

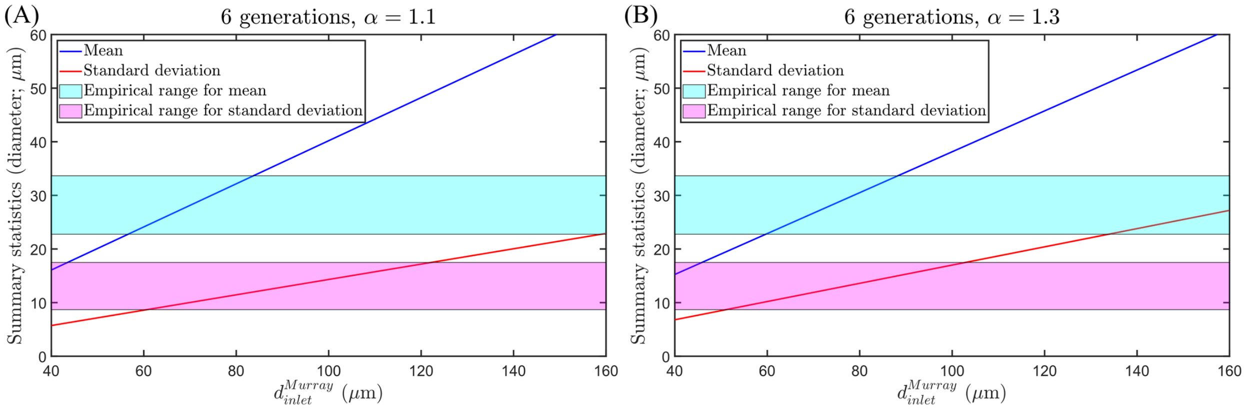

Fig J: The means (blue) and standard deviations (red) of diameter distributions in Murray's law forking networks with 6 **generations of vessels as a function of the inlet diameter (**d_{inlet}^{Murray}**) for (A)** $\alpha = 1.1$ **and (B)** $\alpha = 1.3$**.**

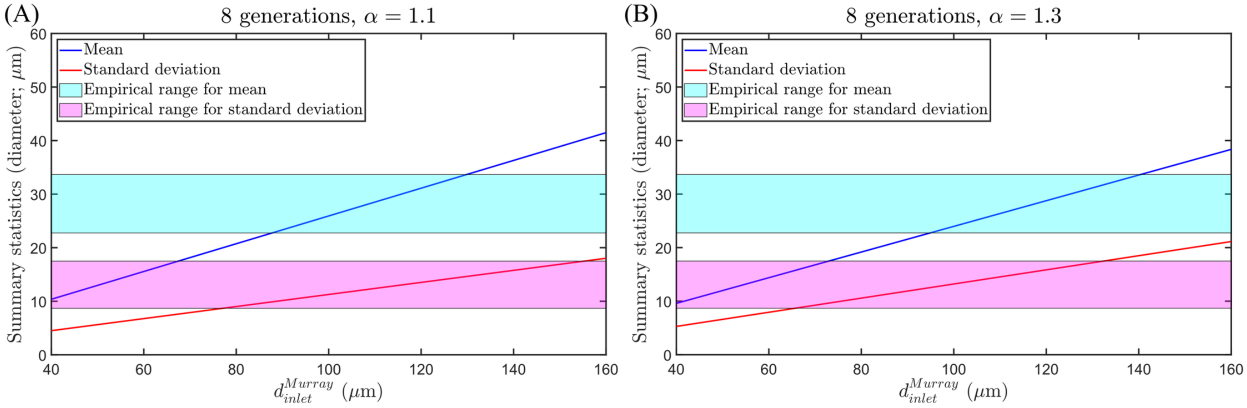

Fig K: The means (blue) and standard deviations (red) of diameter distributions in Murray's law forking networks with 8 **generations of vessels as a function of the inlet diameter (**d_{inlet}^{Murray}**) for (A)** $\alpha = 1.1$ **and (B)** $\alpha = 1.3$**.**

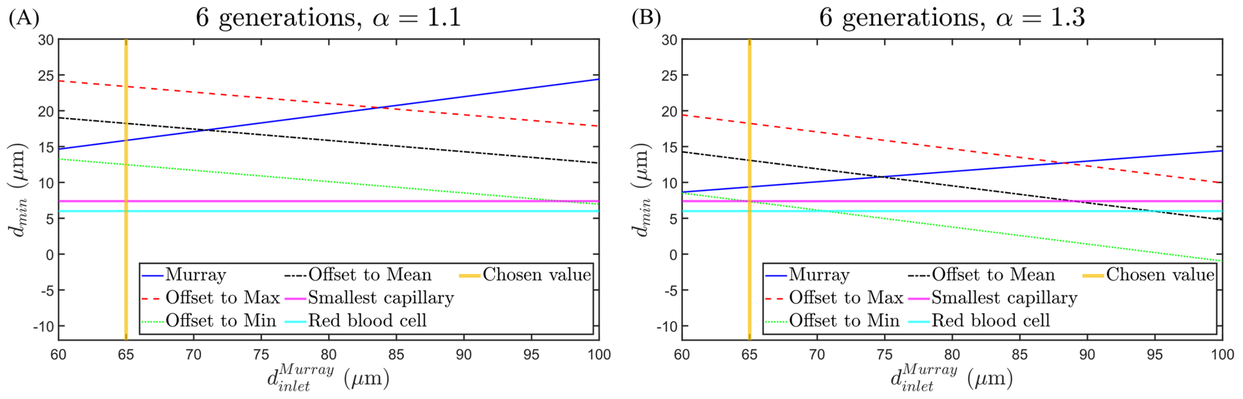

Fig L: The blue curves show minimum vessel diameter (d_{min}**) as a function of inlet diameter (**d_{inlet}^{Murray}**) in Murray's law forking network with** 6 **generations of vessels for (A)** $\alpha = 1.1$ **and (B)** $\alpha = 1.3$**.** The minimum diameter changes due to offsetting to match the smallest (dotted green), average (dash-dotted black), and largest (dashed red) mean diameter in real vasculatures. The diameter of the smallest vessel found in real vasculatures (≈ 7.39 µm) is indicated with horizontal magenta and the minimum diameter of red blood cells (6 µm) with horizontal cyan lines. The value of inlet diameter chosen for our simulations with 6 vessel generations (65 µm) is indicated with vertical yellow lines.

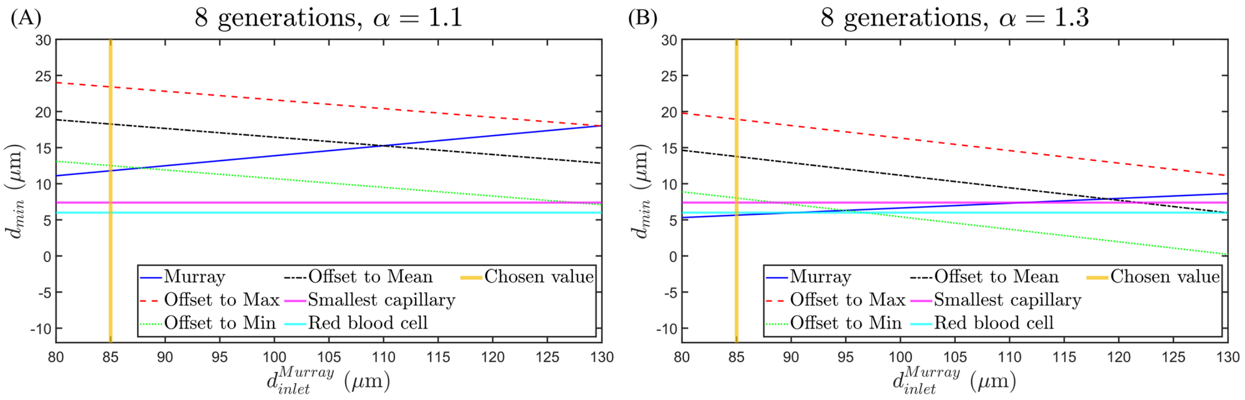

Fig M: The blue curves show minimum vessel diameter (d_{min}) as a function of inlet diameter (d_{inlet}^{Murray}) in Murray's law forking network with 8 generations of vessels for (A) $\alpha = 1.1$ and (B) $\alpha = 1.3$. The minimum diameter changes due to offsetting to match the smallest (dotted green), average (dash-dotted black), and largest (dashed red) mean diameter in real vasculatures. The diameter of the smallest vessel found in real vasculatures (≈ 7.39 µm) is indicated with horizontal magenta and the minimum diameter of red blood cells (6 µm) with horizontal cyan lines. The value of inlet diameter chosen for our simulations with 8 vessel generations (85 µm) is indicated with vertical yellow lines.

Table D: Parameter values for the hexagonal network architecture.

Parameter	Description	Value	Unit	Reference
σ	SD of diameter distribution	8.68, 13.23, 17.49	µm	Section S1.3
d_{min}	minimum diameter	7.39	µm	Section S1.3
d_{max}	maximum diameter	75	µm	Section S1.3
L_{hex}	vessel length	100	µm	Section S1.3
Q_{min}	perfusion threshold	3 x 10^{-13}	m^3 s^{-1}	Section **Perfusion threshold**

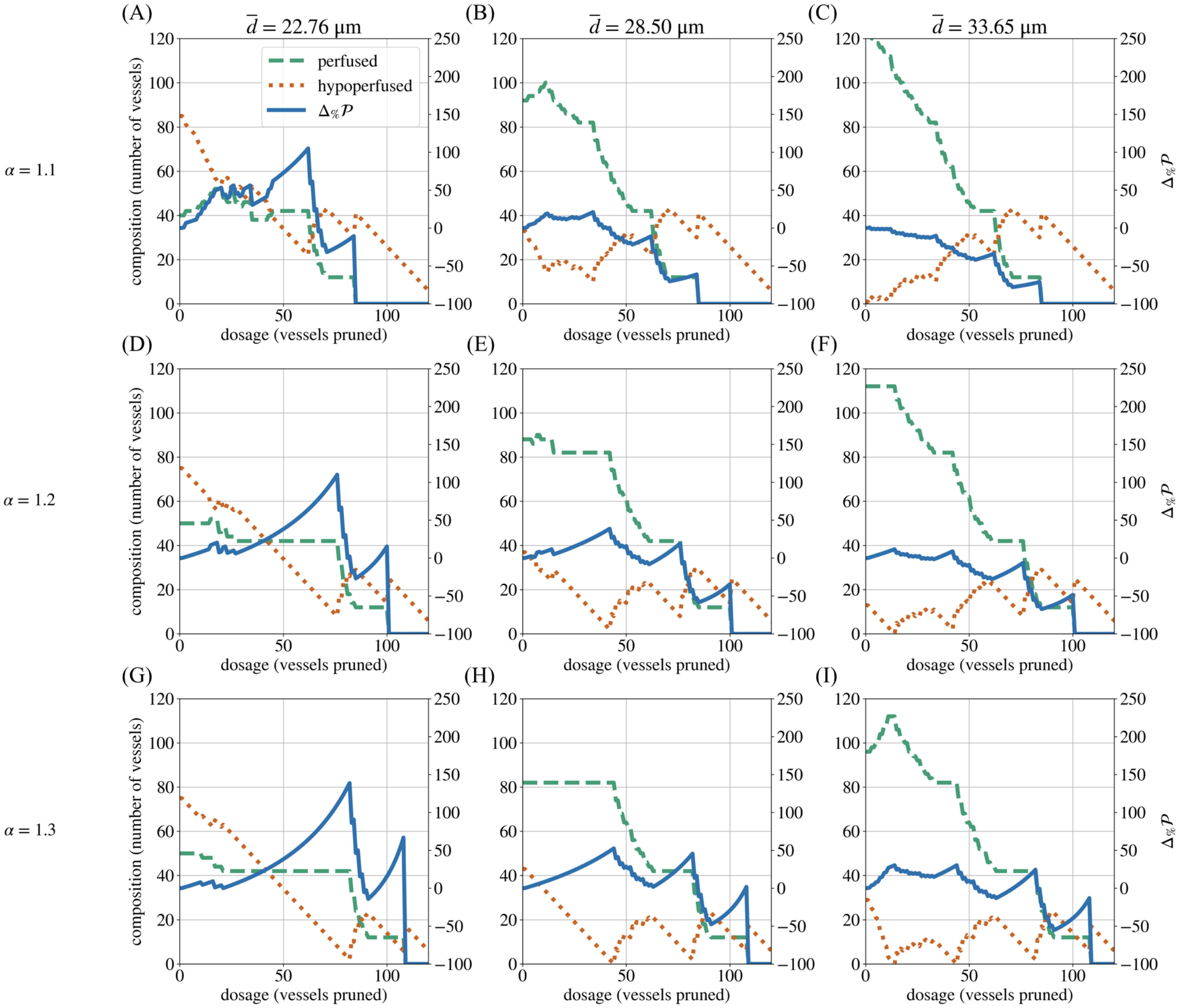

Fig N: A network with 6 generations has the inlet vessel diameter $d_{inlet} = 65$ µm, but has fewer vessels offering resistance to the flow of blood (A–I).

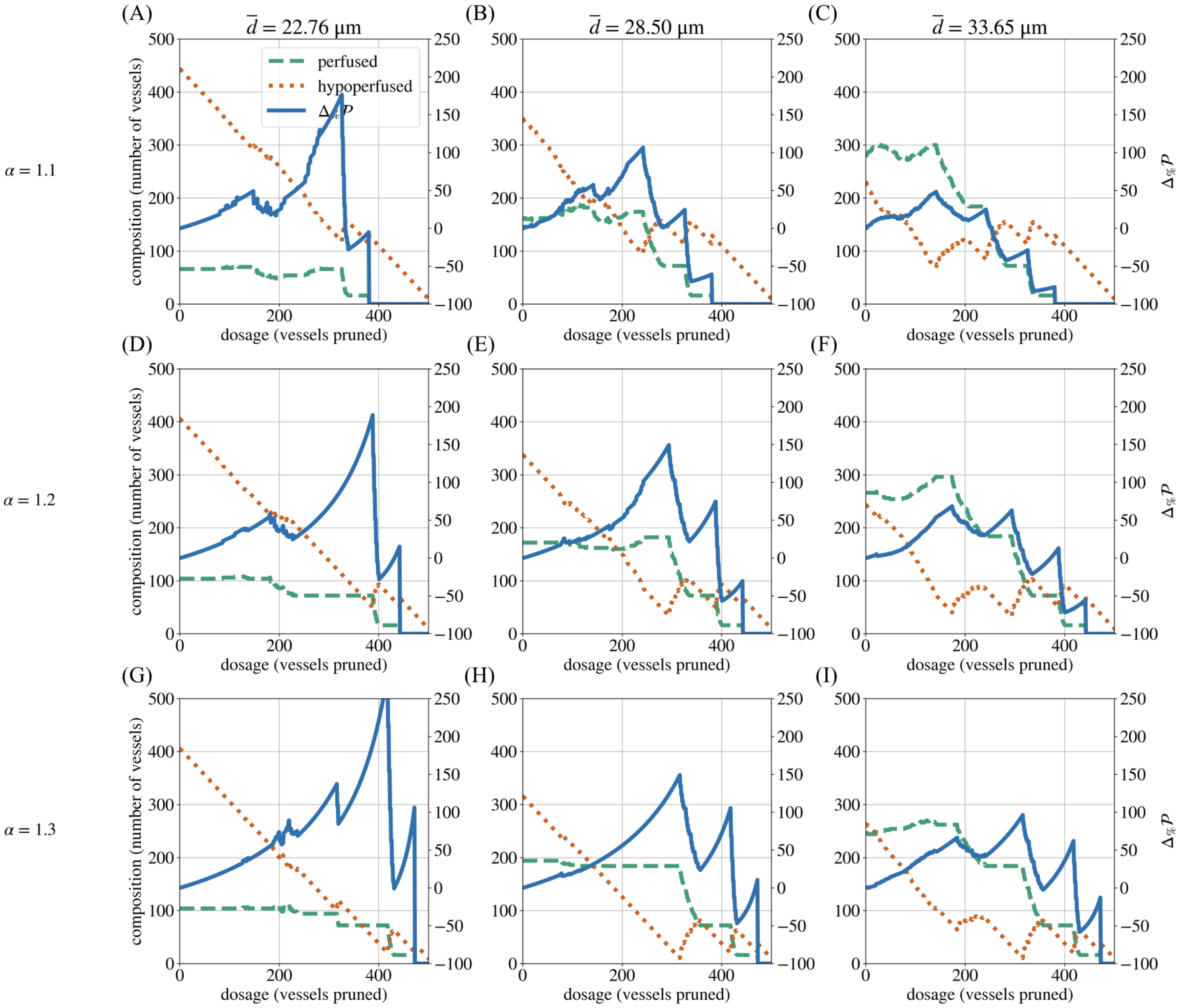

Fig O: A network with 8 generations has the inlet vessel diameter $d_{inlet} = 85$ μm, but has more vessels offering resistance to the flow of blood. The additional hypoperfused vessels allow for a greater increase in PF when they are pruned (A–I).

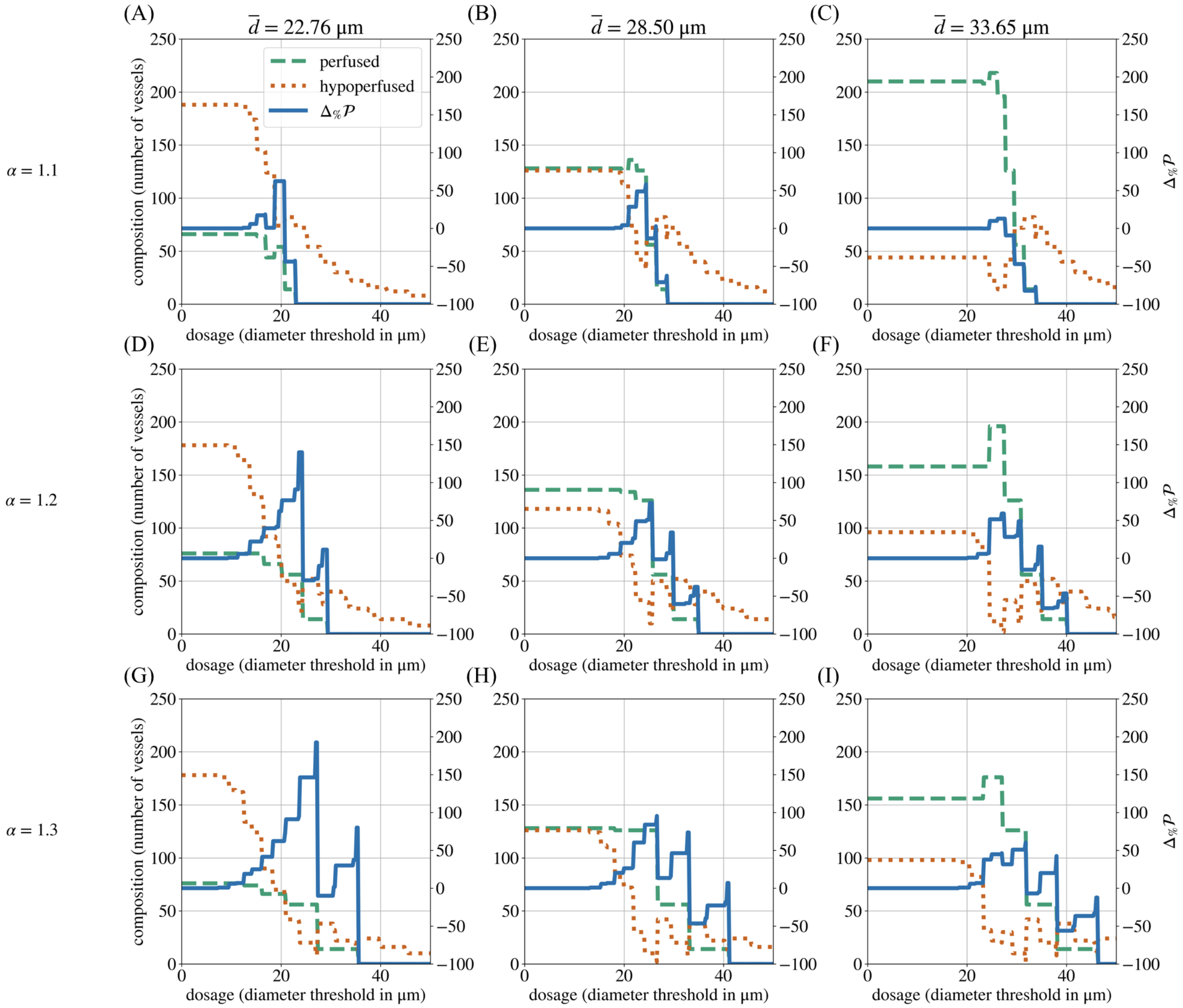

Fig P: Pruning a network with a diameter threshold results in trends similar to those observed when pruning vessels individually (A–I).

References

[1] Bernabeu MO, Köry J, Grogan JA, Markelc B, Beardo A, d'Avezac M, et al. Abnormal morphology biases hematocrit distribution in tumor vasculature and contributes to heterogeneity in tissue oxygenation. Proceedings of the National Academy of Sciences of the United States of America. 2020;117(45):27811–27819. doi:10.1073/pnas.2007770117.

[2] Treuting PM, Dintzis S, Montine KS. Comparative Anatomy and Histology: A Mouse, Rat, and Human Atlas. Academic Press; 2017.

[3] Zhou Q, Perovic T, Fechner I, Edgar LT, Hoskins PR, Gerhardt H, et al. Association between erythrocyte dynamics and vessel remodelling in developmental vascular networks. Journal of the Royal Society Interface. 2021;18(179):20210113.

[4] Billett HH. Hemoglobin and Hematocrit. In: Walker HK, Hall WD, Hurst JW, editors. Clinical Methods: The History, Physical, and Laboratory Examinations. 3rd ed. Boston: Butterworths; 1990. p. 718. Available from: `http://www.ncbi.nlm.nih.gov/books/NBK259/`.

[5] Owen MR, Stamper IJ, Muthana M, Richardson GW, Dobson J, Lewis CE, et al. Mathematical Modeling Predicts Synergistic Antitumor Effects of Combining a Macrophage-Based, Hypoxia-Targeted Gene Therapy with Chemotherapy. Cancer Research. 2011;71(8):2826–2837. doi:10.1158/0008-5472.CAN-10-2834.
